# Identifying inconsistency in network meta‐analysis: Is the net heat plot a reliable method?

**DOI:** 10.1002/sim.8383

**Published:** 2019-10-24

**Authors:** Suzanne C. Freeman, David Fisher, Ian R. White, Anne Auperin, James R. Carpenter

**Affiliations:** ^1^ MRC Clinical Trials Unit at UCL London UK; ^2^ Department of Health Sciences University of Leicester, University Road Leicester UK; ^3^ Meta‐Analysis Platform, Biostatistics and Epidemiology unit Gustave Roussy and INSERM U1018 Levallois‐Perret France; ^4^ London School of Hygiene and Tropical Medicine London UK

**Keywords:** inconsistency, net heat plot, network meta‐analysis

## Abstract

One of the biggest challenges for network meta‐analysis is inconsistency, which occurs when the direct and indirect evidence conflict. Inconsistency causes problems for the estimation and interpretation of treatment effects and treatment contrasts. Krahn and colleagues proposed the net heat approach as a graphical tool for identifying and locating inconsistency within a network of randomized controlled trials. For networks with a treatment loop, the net heat plot displays statistics calculated by temporarily removing each design one at a time, in turn, and assessing the contribution of each remaining design to the inconsistency. The net heat plot takes the form of a matrix which is displayed graphically with coloring indicating the degree of inconsistency in the network. Applied to a network of individual participant data assessing overall survival in 7531 patients with lung cancer, we were surprised to find no evidence of important inconsistency from the net heat approach; this contradicted other approaches for assessing inconsistency such as the Bucher approach, Cochran's Q statistic, node‐splitting, and the inconsistency parameter approach, which all suggested evidence of inconsistency within the network at the 5% level. Further theoretical work shows that the calculations underlying the net heat plot constitute an arbitrary weighting of the direct and indirect evidence which may be misleading. We illustrate this further using a simulation study and a network meta‐analysis of 10 treatments for diabetes. We conclude that the net heat plot does not reliably signal inconsistency or identify designs that cause inconsistency.

## 1 INTRODUCTION

Network meta‐analysis (NMA) is an extension of pairwise meta‐analysis methods that combines direct and indirect evidence from a network of trials to calculate a treatment effect for every treatment comparison within a single statistical model. A key assumption of NMA is the consistency of direct and indirect evidence. Consistency equations were first set out by Higgins and Whitehead^1^ who showed that the relative effects of different treatments could be jointly estimated by “borrowing strength” from direct comparisons to inform indirect comparisons. Inconsistency in NMA occurs when the direct and indirect evidence are not in agreement with each other. This can result in biased treatment effect estimates. Inconsistency within a network may arise when bias in direct comparisons (for example optimism bias, publication bias or sponsorship bias) acts differently in different comparisons or when treatment effect modifiers are distributed differently in different comparisons.^2^, ^3^ The power of tests for inconsistency is generally low because indirect evidence is typically a relatively weak component of most treatment estimates in NMA. Failure to reject the null hypothesis of no inconsistency does not mean that the entire network is consistent.^4^ Nevertheless,the increasing use of NMA in health decision modeling means that it is important that attempts are made to identify, understand, and where appropriate, adjust for inconsistency.

As is typical in the NMA literature, throughout this paper, “design” will refer to the treatments being compared within a trial.^5^ For example, two trials both comparing treatment A to treatment B will be considered to be of the same design, whereas a third trial comparing treatment A to treatment B and treatment C will be considered to be of a different design. For a full review of NMA methods, see Salanti^6^ and Efthimiou et al.^7^

There are several approaches for assessing inconsistency in a network; in particular, we take a closer look at Cochran's Q statistic,^8^ the loop inconsistency approach,^9^ the inconsistency parameter approach,^10^ node‐splitting,^11^ and the net heat approach.^12^ Between them, these five methods offer a range of increasingly complex methods for identifying inconsistency in a network. Cochran's Q statistic^8^ and the loop inconsistency approach of Bucher^9^ are relatively simple methods that aim to identify inconsistency through one test statistic and a p‐value. Both the inconsistency parameter approach of Lu and Ades^10^ and node‐splitting^11^ allow for inconsistency in a Bayesian hierarchical model, which allows the amount of inconsistency to be quantified and a credible interval calculated. Krahn et al^12^ also use a modeling approach; however, the results are displayed graphically as a net heat plot, with the aim of allowing inconsistency to be identified, and are not linked to a statistical test.

Cochran's Q statistic^8^ is a common method for assessing heterogeneity in a meta‐analysis. The generalized Cochran's Q statistic for multivariate meta‐analyses^13^ can be used in the context of NMA to quantify heterogeneity across the whole network, both within trial designs and between trial designs (the latter is known as inconsistency).

Bucher^9^ developed a method for assessing loop inconsistency in loops of three treatments within a network consisting of two‐arm trials only. The approach involves calculating the difference between the direct and indirect evidence for a treatment comparison and testing it against the null hypothesis of consistency by referring the test statistic to the normal distribution. However, in a large network where each treatment loop is considered one at a time, multiple testing must be taken into account, and this approach can be both cumbersome and time consuming.^14^, ^15^

One of the most popular models to account for inconsistency in a network is the Bayesian hierarchical model of Lu and Ades.^10^ This model is a generalization of the Bucher approach and relaxes the consistency assumption by including an inconsistency parameter in each loop in which inconsistency could occur. These additional inconsistency parameters can be fitted as fixed or random effects. Models with and without inconsistency parameters are then compared to assess whether a network is consistent and the analyst must make an arbitrary choice about this. However, in the presence of multi‐arm trials, this approach depends on the order of treatments.

Cochran's Q statistic,^8^ the loop inconsistency approach,^9^ and the inconsistency parameter approach^10^ all provide a global assessment of inconsistency in a network; however, local methods for assessing inconsistency are also needed in order to identify which treatment comparisons are driving the inconsistency.^11^ Dias et al^11^ first proposed comparison‐specific assessment of inconsistency using node‐splitting. Node‐splitting involves separating out the evidence for a particular treatment comparison into the direct and indirect evidence and assessing the discrepancy between them, one treatment comparison at a time.^11^ Node‐splitting can be considered equivalent to the inconsistency parameter approach of Lu and Ades if all the treatment nodes are split at the same time so that separate treatment effects are estimated for each treatment comparison without assuming consistency over any set of trials.^11^

To aid the identification of inconsistency within a network, Krahn et al^12^ developed a method, known as the net heat plot, which could be used as a visual aid for locating and identifying any inconsistency within a network of randomized controlled trials (RCTs). The net heat plot uses Cochran's Q statistic in a fixed effect framework and decomposes it into within‐trial heterogeneity and inconsistency. The net heat plot is constructed by temporarily removing each design one at a time and assessing the contribution of each design to the inconsistency of the whole network. The difference between the inconsistency in the network before the temporary removal of each design and the inconsistency that remains following the temporary removal of each design, known as *Q* ^diff^, is displayed graphically in the form of a matrix. The net heat plot is then colored so that the coloring of each square indicates designs which increase or decrease inconsistency within the network.

Cochran's Q statistic, the loop inconsistency approach, the inconsistency parameter approach, and node‐splitting all use formal statistical tests to draw conclusions about possible inconsistency in a network. In contrast, *Q* ^diff^ (the difference between two Q statistics, which themselves follow chi‐squared distributions) has a nonstandard distribution and is therefore much harder to interpret. The coloring of the net heat plot is driven by *Q* ^diff^, and it is unclear what value of *Q* ^diff^ constitutes statistically significant or clinically meaningful inconsistency.

In this paper, we take a closer look at the net heat plot and highlight some previously unremarked limitations of this approach. In Section 2, we introduce two networks of trials in lung cancer and diabetes and assess the possibility of inconsistency using a visual approach. In Section 3, we consider five methods for assessing inconsistency in NMA: Cochran's Q statistic,^8^ the loop inconsistency approach,^9^ the inconsistency parameter approach,^10^ node‐splitting,^11^ and the net heat plot.^12^ In Section 4, we derive algebraic expressions for the elements of the net heat plot in terms of direct treatment estimates and interpret them with the aid of numerical simulations in Section 5. In Section 6, we apply the five methods of assessing inconsistency to the lung cancer and diabetes networks before offering a conceptual critique in Section 7. In Section 8, we finish with a discussion.

## 2 DATASETS

In this section, we introduce two datasets to which we will apply methods for assessing inconsistency in NMA. We first introduce a simple three‐treatment network for lung cancer (to illustrate the underlying arguments) and secondly a more complex network of 10 treatments for diabetes.

### 2.1 Lung cancer network

**Figure 1:**
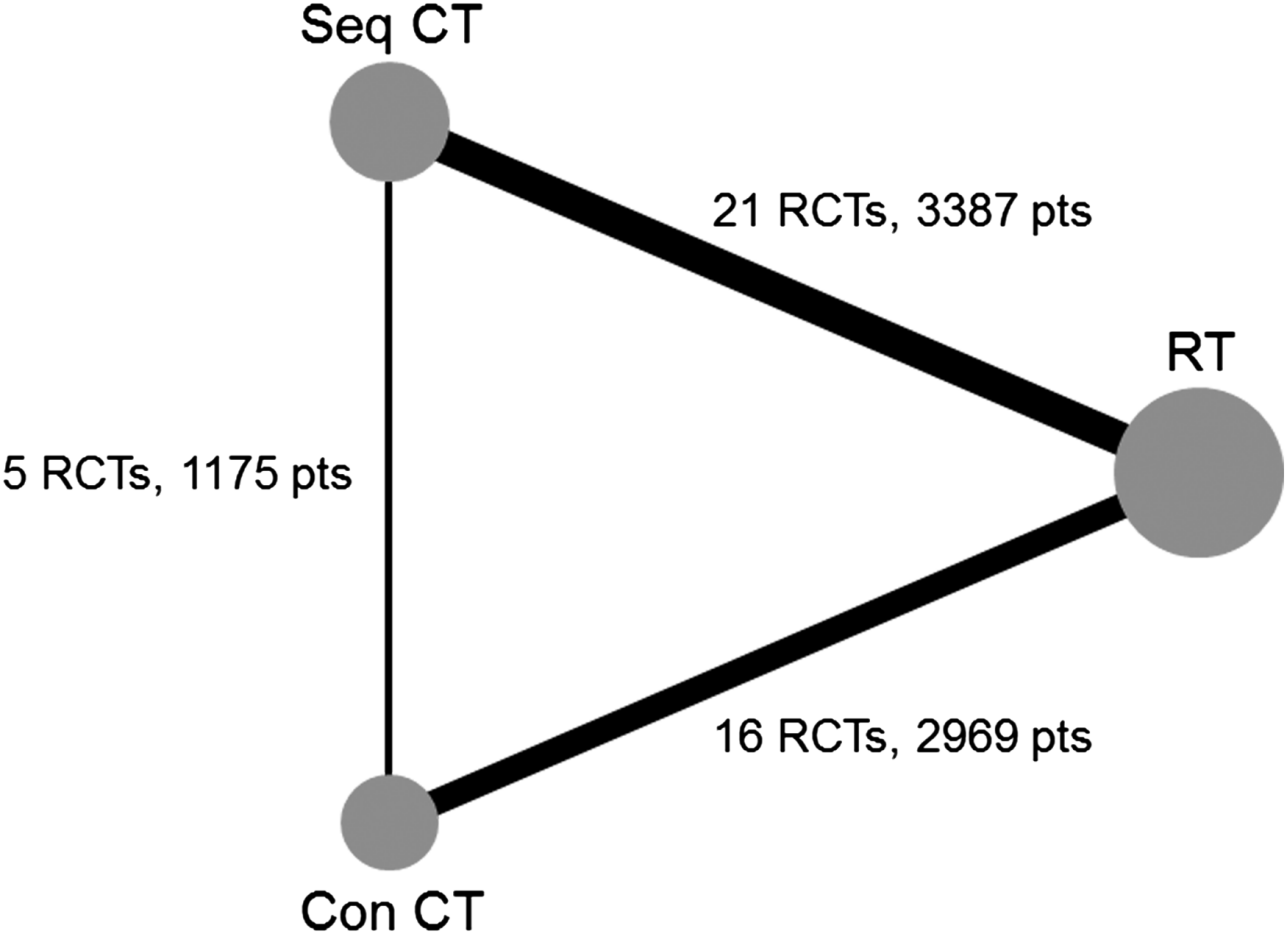
Lung cancer network diagram. The node size is weighted according to the number of patients randomized to each treatment, and the line thickness is weighted according to the number of studies involved in each direct comparison. Key to treatments: Con CT, radiotherapy plus concomitant chemotherapy; Pts, patients;RCTs, randomized clinical trials; RT, radiotherapy; Seq CT, radiotherapy plus sequential chemotherapy

For our first network, we consider the simplest network structure possible: one treatment loop consisting of three treatments without multiarm trials. The data for this network come from three meta‐analyses of RCTs in lung cancer performed by the Non‐Small‐Cell Lung Cancer Collaborative Group. These data were obtained from Gustave‐Roussy (GR), Paris. The three meta‐analyses considered three different treatments: radiotherapy (RT), radiotherapy plus sequential chemotherapy (Seq CT), and radiotherapy plus concomitant chemotherapy (Con CT) using three different designs: RT v Seq CT, RT v Con CT, and Seq CT v Con CT (Figure 1).

The meta‐analysis (MA) of RT and Seq CT was published in 1995 and included 3033 patients from 22 RCTs.^16^ The current dataset was updated by GR to include some newer trials and exclude some trials using older forms of chemotherapy. This comparison now includes a total of 21 RCTs and 3387 patients. The MA of RT and Con CT was published in the work of Auperin et al^17^ and included 1764 patients from 9 RCTs. This MA was also updated by GR to include a total of 16 trials and 2969 patients. The MA of Seq CT and Con CT was published in 2010 and included 6 RCTs and 1205 patients.^18^ One multiarm trial (45 patients) comparing all three treatments was excluded from the network for the analyses in this paper in order to obtain the simplest network structure possible for a network meta‐analysis. In total, overall survival data was available for 7531 patients from 42 RCTs. A list of all RCTs is provided in Appendix A (supplementary material).

The lung cancer network forms one treatment loop, so there can only be one inconsistency source. It provides a simple yet revealing starting point for assessing the net heat plot. To visually assess the agreement between the direct and indirect evidence within the lung cancer network, before any formal statistical models were fitted, the treatment effects for all pairwise comparisons were estimated in a number of ways. Network estimates combining both direct and indirect treatment effects were obtained by fitting a one‐step IPD NMA Royston‐Parmar model for time‐to‐event data^19^, ^20^ using a Bayesian approach and by fitting a two‐step NMA using the R package netmeta.^21^ An estimate of the direct evidence was obtained by fitting the one‐step IPD Royston‐Parmar MA model to trials directly comparing the treatments of interest only. Indirect treatment effects were also calculated using the one‐step IPD Royston‐Parmar MA model, where all trials directly comparing the two treatments of interest were excluded from the model. Throughout this paper, all models are fitted with fixed effects assuming no heterogeneity in any of the direct comparisons to simplify calculations in later sections of the paper. In the Bayesian estimation of the Royston‐Parmar model, parameters representing the spline function for the baseline log cumulative hazard function and treatment effects were fitted with noninformative normal prior distributions.

**Figure 2:**
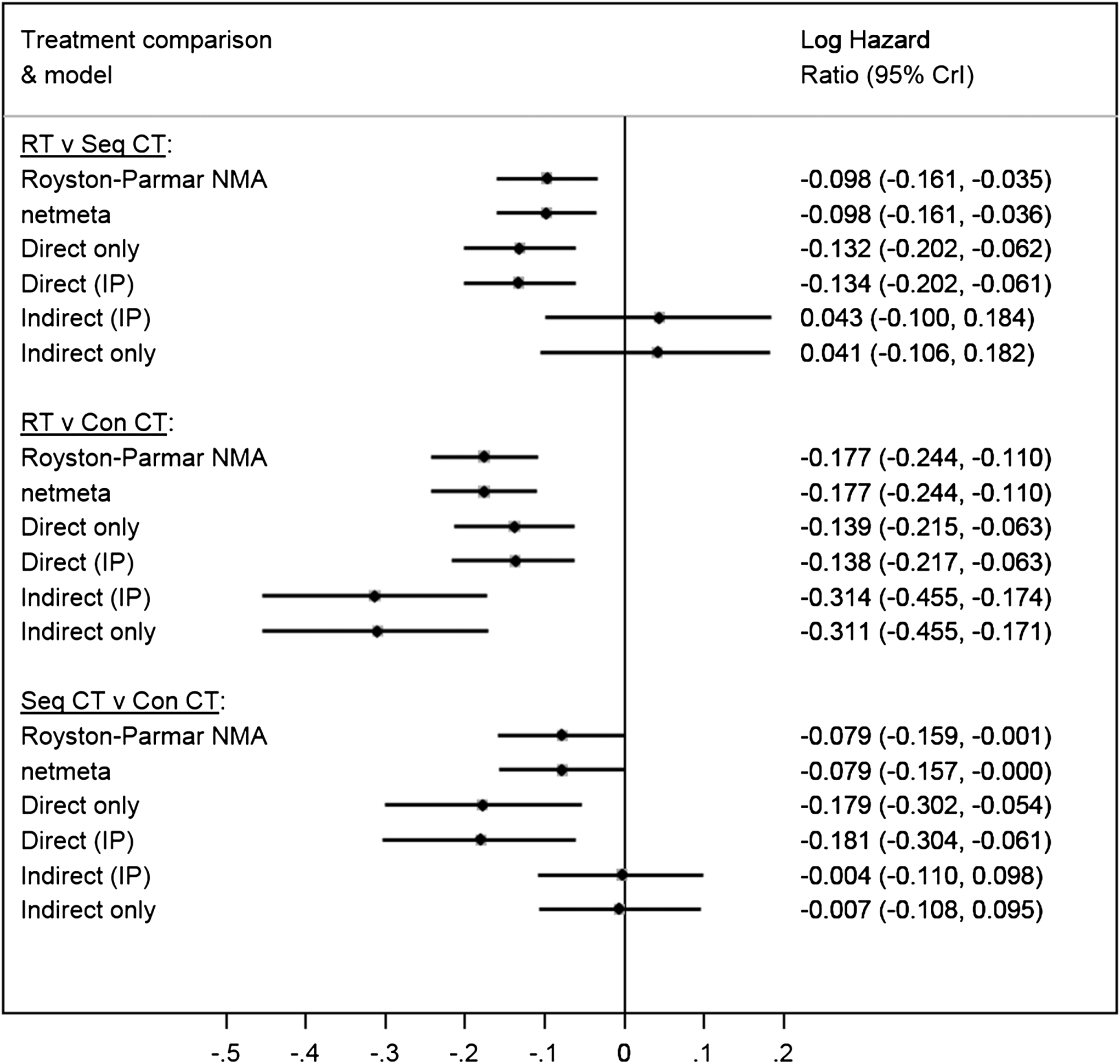
Forest plot of various analyses of the lung cancer data. All models were fitted with fixed effects. Key to treatments: Con CT, concomitant chemotherapy; CrI, credible interval (except netmeta models where confidence intervals are presented); IP, inconsistency parameter; NMA, network meta‐analysis; RT, radiotherapy; Seq CT, sequential chemotherapy

Figure 2 presents the forest plot of treatment effects for each pairwise comparison, using the methods described above and including the results of the inconsistency parameter approach, described below in Section 3.3. The forest plot clearly shows a difference between the direct and indirect evidence for each pairwise comparison.

### 2.2 Diabetes network

**Figure 3:**
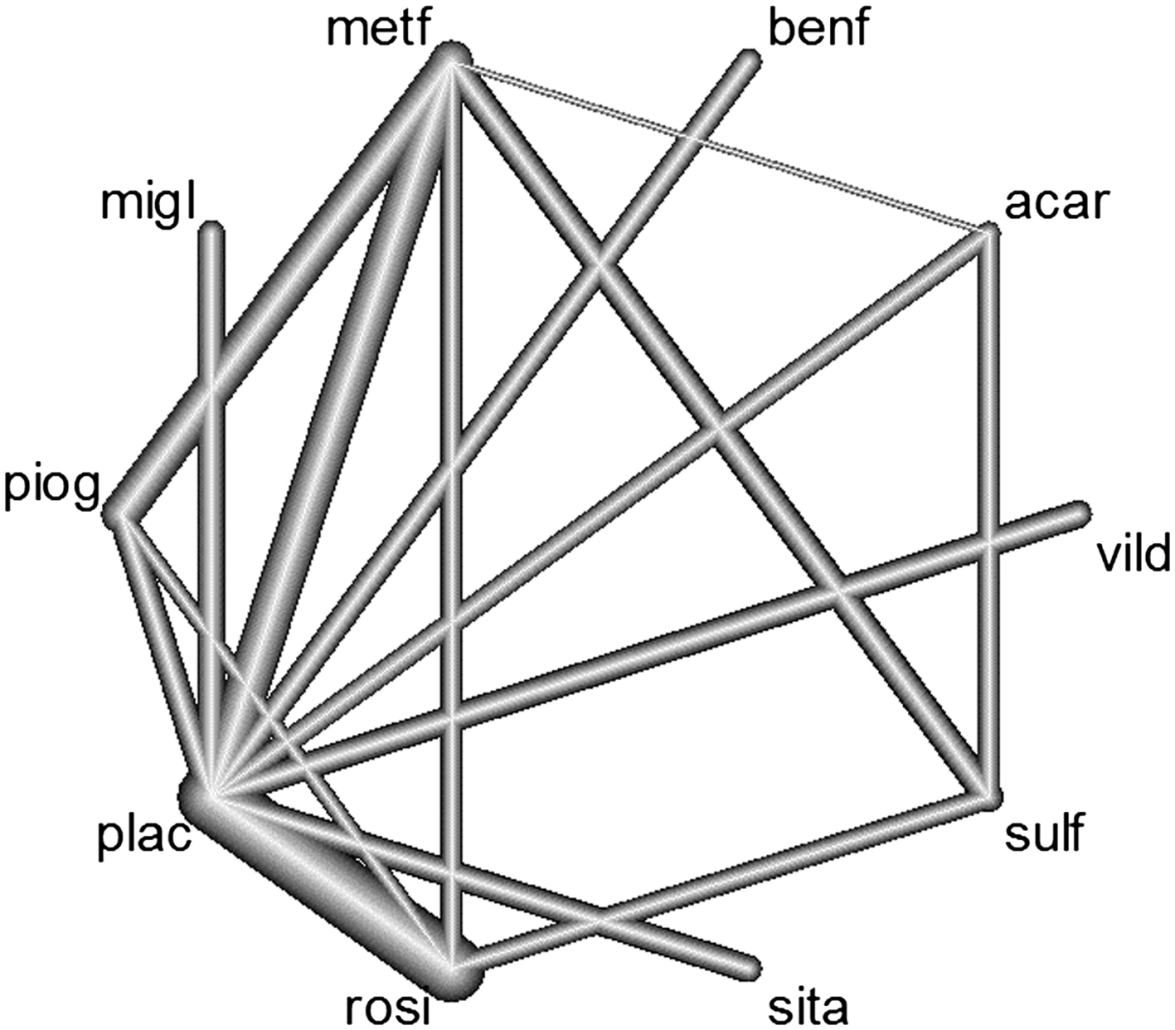
Diabetes network diagram. The line thickness is proportional to the number of studies involved in each comparison. Multiarm trial includes placebo, metformin and acarbose. Key to treatments: acar, acarbose; benf, benfluorex; metf, metformin; migl, miglitol; piog, pioglitazone; plac, placebo; rosi, rosiglitazone; sita, sitagliptin; sulf, sulfonylurea; vild, vildagliptin

For our second network, we consider a more complex network structure consisting of multiple treatments and multiarm trials. The network considers 10 treatments for type 2 diabetes: acarbose (acar), benfluorex (benf), metformin (metf), miglitol (migl), pioglitazone (piog), placebo (plac), rosiglitazone (rosi), sitagliptin (sita), sulfonylurea alone (sual), vildaglitin (vild) using 15 different designs: metf v plac, acar v metf v plac, piog v plac, metf v piog, piog v rosi, metf v rosi, rosi v sual, acar v sual, acar v plac, plac v vild, metf v sual, migl v plac, metf v rosi, migl v rosi, benf v plac (Figure 3). The data for this network were initially collected and reported by Senn et al.^22^ In total, glycated haemoglobin (HbA1c) data were available for 6646 patients from 26 RCTs.

The diabetes network contains multiple treatment loops and provides a more challenging example for assessing inconsistency. To visually assess the agreement between the direct and indirect evidence within the diabetes network, we fitted a two‐step NMA using the R package netmeta^21^ and obtained estimates of the direct and indirect evidence from node‐splitting.

Figure S1 (supplementary material) presents the forest plot of treatment effects for each pairwise comparison. The forest plot clearly shows a difference between the direct and indirect evidence for the pairwise comparisons of metf v sulf and rosi v sulf.

## 3 METHODS FOR ASSESSING INCONSISTENCY IN NMA

In this section, we describe five methods for assessing inconsistency in NMA.

### 3.1 Cochran's Q statistic

Cochran's *Q* statistic can be used to assess heterogeneity within a network. The overall *Q* statistic from the fixed effect NMA model can be decomposed into within‐design heterogeneity (*Q* ^het^) and between‐design heterogeneity, which is termed design inconsistency (*Q* ^inc^). Let ${\hat{\theta }}_{ic}$ be the treatment effect estimate from trial *i* for the comparison of treatments in design *c* with corresponding standard error ${\hat{\sigma }}_{ic}$, where there are 1,…,*n*_*c*_ trials of design *c*. Let ${\hat{\theta }}_{c}$ be the treatment effect from the direct evidence for design *c* only with corresponding standard error ${\hat{\sigma }}_{c}$ and ${\hat{\theta }}_{Nc}$ be the network estimate of the treatment effect for design *c*; then,

$$Q=\underset{c}{\sum }\overset{n_{c}}{\underset{i=1}{\sum }}{\left\{\frac{{\hat{\theta }}_{ic}-{\hat{\theta }}_{Nc}}{{\hat{\sigma }}_{ic}}\right\}}^{2}$$

$$\;Q^{\text{het}}=\underset{c}{\sum }\overset{n_{c}}{\underset{i=1}{\sum }}{\left\{\frac{{\hat{\theta }}_{ic}-{\hat{\theta }}_{c}}{{\hat{\sigma }}_{ic}}\right\}}^{2}$$

$$\;Q^{\text{inc}}=\underset{c}{\sum }{\left\{\frac{{\hat{\theta }}_{c}-{\hat{\theta }}_{Nc}}{{\hat{\sigma }}_{c}}\right\}}^{2},$$

with *Q*=*Q* ^het^+*Q* ^inc^.

For multiarm studies, ${\hat{\theta }}_{ic}$ is a vector with variance *S*_*ic*_, and these formulae are extended to $\sum_{c}\sum_{i}{\left({\hat{\theta }}_{ic}-{\hat{\theta }}_{Nc}\right)}^{T}S_{ic}^{-1}({\hat{\theta }}_{ic}-{\hat{\theta }}_{Nc})$, etc.^13^

### 3.2 Loop inconsistency

From now on, throughout this paper, we use the shorthand *dir* to represent direct evidence, *ind* to represent indirect evidence and *net* to represent network evidence (ie, the combination of the direct and indirect evidence). In a loop of three treatments A, B, and C, we compared the direct evidence of treatment C versus treatment A, ${\hat{\theta }}_{AC}^{\text{dir}}$, to the indirect evidence, ${\hat{\theta }}_{AC}^{\text{ind}}$, where ${\hat{\theta }}_{AC}^{\text{ind}}={\hat{\theta }}_{AB}^{\text{dir}}+{\hat{\theta }}_{BC}^{\text{dir}}$ and $\mathrm{Var}({\hat{\theta }}_{AC}^{\text{ind}})=\mathrm{Var}({\hat{\theta }}_{AB}^{\text{dir}})+\mathrm{Var}({\hat{\theta }}_{BC}^{\text{dir}})$. Following the method of Bucher,^9^ estimates of the inconsistency parameter, ${\hat{\omega }}_{AC}$, and its variance can be formed, within a loop, by subtracting the direct and indirect estimates

(1) $$\;{\hat{\omega }}_{AC}={\hat{\theta }}_{AC}^{\text{dir}}-{\hat{\theta }}_{AC}^{\text{ind}}$$

$$\mathrm{Var}({\hat{\omega }}_{AC})=\mathrm{Var}\left({\hat{\theta }}_{AC}^{\text{dir}}\right)+\mathrm{Var}\left({\hat{\theta }}_{AC}^{\text{ind}}\right)=\mathrm{Var}\left({\hat{\theta }}_{AC}^{\text{dir}}\right)+\mathrm{Var}\left({\hat{\theta }}_{AB}^{\text{dir}}\right)+\mathrm{Var}\left({\hat{\theta }}_{BC}^{\text{dir}}\right).$$

An approximate test of the null hypothesis of consistency is conducted by referring the test statistic $z_{AC}=\frac{{\hat{\omega }}_{AC}}{\sqrt{\mathrm{Var}({\hat{\omega }}_{AC})}}$ to the normal distribution.

### 3.3 Inconsistency parameter approach

The inconsistency parameter approach of Lu and Ades^10^ involves adding an extra parameter (the inconsistency parameter) to each treatment loop within a network to assess inconsistency and estimate both the direct and indirect evidence simultaneously. This allows estimates of the direct and indirect information to be obtained for each comparison within the treatment loop. In a network containing one three‐treatment loop between treatments A, B, and C, let *ω*_*ABC*_ represent the inconsistency parameter for this loop. For example, under the Royston‐Parmar model for time‐to‐event outcomes, the log cumulative hazard for patient *i* in trial *j* is given by

(2) $$\ln\{H_{ij}(t|x_{ij})\}=s_{j}\left(\ln(t)\right)+\beta_{1}{\text{trt1}}_{ij}+\beta_{2}{\text{trt2}}_{ij}-\omega_{ABC}{\text{trt1}}_{ij}{\text{trt2}}_{ij},$$

where $s_{j}(\ln(t))$ is the restricted cubic spline modeling the baseline log cumulative hazard for trial *j*, trt1_*ij*_ and trt2_*ij*_ are treatment indicator variables, and *β*_1_ and *β*_2_ are the treatment effect estimates for trt1_*ij*_ and trt2_*ij*_ compared to the reference treatment, respectively.

### 3.4 Node‐splitting

Node‐splitting compares a model where the consistency assumption is relaxed for one treatment comparison to the model assuming consistency across the entire network to highlight inconsistent treatment comparisons within the network. Each treatment comparison is considered separately and one at a time for evidence of possible inconsistency. Node‐splitting can be implemented using the “network sidesplit all” command^23^ in Stata,^24^ which reports the treatment effects from the direct and indirect evidence together with their difference and a test of whether the true difference is equal to zero for each treatment comparison.^23^

### 3.5 Net heat plot

In 2013, Krahn et al^12^ introduced the net heat plot as a method for identifying and locating inconsistency within a network of RCTs. In a network of RCTs with at least one treatment loop, the net heat plot is constructed by temporarily removing (also referred to as detaching) each design one at a time and assessing the contribution of each design to the inconsistency of the whole network.

Krahn et al^12^ propose the use of a design‐by‐treatment interaction approach, whereby the consistency assumption for one of the treatment loops is relaxed so that the remaining inconsistency across the network can be calculated. In practice, this is computationally simple because it is equivalent to a “leave one out” approach in which *Q* ^inc^ is simply recalculated from scratch after the (temporary) removal of each design in turn (which is equivalent to removing each loop in turn, assuming each design features in only one loop). Designs that do not contribute to a treatment loop or when removed would split the network into two distinct parts are excluded from the net heat plot.

In an NMA model, the design matrix contains the structure of the network at the study level and links the observed treatment effects with the treatment contrast parameters. To detach design *d*, we add to the design matrix additional columns. The number of columns to add is equal to the number of treatments in design *d* minus 1. Thus, when design *d* includes two treatments, one column is added, consisting of a “1” in the row corresponding to the design, which is being detached and “0” elsewhere (this is analogous to perfectly fitting an observation in a regression by including a dummy variable for just that observation). The treatment effects for each comparison in the network are then recalculated using this new design matrix, and the inconsistency in the network when design *d* is detached is thus calculated.

The between‐design inconsistency statistic, *Q* ^inc^, is the part of the total heterogeneity in the network that is not explained by heterogeneity within designs. Let $Q_{c}^{\text{inc}}$ represent the inconsistency in the network for design *c* before any designs are detached, where $Q^{\text{inc}}=\sum_{c}Q_{c}^{\text{inc}}=\sum_{c}{\left({\hat{\theta }}_{c}^{\text{dir}}-X_{c}{\hat{\theta }}^{\text{net}}\right)}^{\prime }\mathrm{Var}{\left({\hat{\theta }}_{c}^{\text{dir}}\right)}^{-1}({\hat{\theta }}_{c}^{\text{dir}}-X_{c}{\hat{\theta }}^{\text{net}})$, where *X*_*c*_ is the design matrix and ${\hat{\theta }}^{\text{net}}$ is the vector of treatment parameter estimates. Let $Q_{c(d)}^{\text{inc}}$ represent the inconsistency remaining in the network for design *c* when design *d* is detached and $Q_{c,d}^{\text{diff}}$ denote the change in inconsistency for design *c* resulting from detaching design *d*. Then,

$$Q_{c,d}^{\text{diff}}=Q_{c}^{\text{inc}}-Q_{c(d)}^{\text{inc}}.$$

The values of $Q_{c,d}^{\text{diff}}$ form the basis of the net heat plot. The net heat plot is constructed as a matrix in which each off‐diagonal square is $Q_{c,d}^{\text{diff}}$, representing the contribution of the row design (*c*) to the total inconsistency across the network when the column design (*d*) is detached (ie, the consistency assumption is relaxed for the column design). The leading diagonal, running from the top left to the bottom right corner, displays the contribution of each design *c*, $Q_{c}^{\text{inc}}$, to the between design statistic, *Q* ^inc^.

Moreover, in each net heat plot, the area of the grey squares within each matrix cell are proportional to the absolute values of the hat matrix (of the NMA regression model with no designs detached). These are interpretable as the (statistical information) contribution of the direct estimate of the column design to the network estimate of the row design. As proposed by Krahn et al, the net heat plot is colored so that values of $Q_{c,d}^{\text{diff}}>0$ take on yellow and red colors and values of $Q_{c,d}^{\text{diff}}<0$ take on white and blue colors. The coloring varies in intensity with the maximum intensity (ie, the brightest colors) representing absolute values of $Q_{c,d}^{\text{diff}}$ greater than or equal to eight. Red colors indicate that the contribution of the evidence from the column design toward the row design is inconsistent with the other evidence in the network. Blue colors indicate that the contribution of the evidence from the column design toward the row design is consistent with the other evidence in the network.^25^ This enables the reader to identify which designs are most likely to be responsible for the inconsistency in the network.

Net heat plots can be produced with the package netmeta^21^ in R.^26^

## 4 A CLOSER LOOK AT THE NET HEAT PLOT

As NMA is a form of regression, we would expect any diagnostic useful in the NMA case to be meaningful in simpler cases. We now look in more detail at the calculation underlying the net heat plot starting in Section 4.1 by considering a three‐treatment network before generalizing the result and exploring the interpretation in Section 4.2.

### 4.1 Three‐treatment network

We consider a three‐treatment network, consisting of treatments A, B, and C, in which direct evidence is available for all pairwise comparisons. In this setting, we consider two‐arm trials only. The aim here is to look at what happens to the inconsistency for design *c* when we detach design *d*. There are two possible scenarios: *d*≠*c* and *d*=*c*.

In a network of three treatments, there is only one pathway of indirect evidence. For example, for the comparison AC, the pathway of indirect evidence goes via treatment B. We denote the direct treatment effect by ${\hat{\theta }}_{c}^{\text{dir}}$ and the indirect treatment effect by ${\hat{\theta }}_{c}^{\text{ind}}$. Applying these definitions to a three‐treatment network, consisting of treatments A, B, and C and letting *c*=*AC*, we have

$$\;{\hat{\theta }}_{c}^{\text{dir}}={\hat{\theta }}_{AC}^{\text{dir}}\text{, with variance}\;s_{AC}^{2},$$

$${\hat{\theta }}_{c}^{\text{ind}}={\hat{\theta }}_{AB}^{\text{dir}}+{\hat{\theta }}_{BC}^{\text{dir}}\text{, with variance}\;s_{AB}^{2}+s_{BC}^{2}.$$

The network estimate is equal to the inverse variance weighted average of all the direct and indirect evidence combined

$${\hat{\theta }}_{AC}^{\text{net}}=\frac{s_{AB}^{2}+s_{BC}^{2}}{s_{AC}^{2}+s_{AB}^{2}+s_{BC}^{2}}{\hat{\theta }}_{AC}^{\text{dir}}+\frac{s_{AC}^{2}}{s_{AC}^{2}+s_{AB}^{2}+s_{BC}^{2}}{\hat{\theta }}_{AC}^{\text{ind}}.$$

For design *c*, the inconsistency Q statistics are defined as

(3) $$Q_{c}^{\text{inc}}=\frac{1}{s_{c}^{2}}{\left({\hat{\theta }}_{c}^{\text{dir}}-{\hat{\theta }}_{c}^{\text{net}}\right)}^{2}$$

(4) $$Q_{c(d)}^{\text{inc}}=\frac{1}{s_{c}^{2}}{\left({\hat{\theta }}_{c}^{\text{dir}}-{\hat{\theta }}_{c(d)}^{\text{net}}\right)}^{2},$$

where $s_{c}^{2}=\mathrm{Var}({\hat{\theta }}_{c}^{\text{dir}})$.

$Q_{c}^{\text{inc}}$ represents the difference between the direct and network evidence for design *c* across the whole network. Continuing with *c*=AC, we have

$$Q_{AC}^{\text{inc}}=\frac{1}{s_{AC}^{2}}{\left({\hat{\theta }}_{AC}^{\text{dir}}-{\hat{\theta }}_{AC}^{\text{net}}\right)}^{2}=\frac{1}{s_{AC}^{2}}{\left[\frac{s_{AC}^{2}}{s_{AC}^{2}+s_{AB}^{2}+s_{BC}^{2}}\left({\hat{\theta }}_{AC}^{\text{dir}}-{\hat{\theta }}_{AC}^{\text{ind}}\right)\right]}^{2}.$$

$Q_{c(d)}^{\text{inc}}$ represents the difference between the direct and network evidence for design *c* when design *d* is detached, and $Q_{c,d}^{\text{diff}}$ represents the change in inconsistency for design *c* when design *d* is excluded from the network so that

(5) $$Q_{AC,d}^{\text{diff}}=Q_{AC}^{\text{inc}}-Q_{AC(d)}^{\text{inc}}.$$

When *d*≠*c*, the pathway of indirect evidence must include design *d*. Therefore, the network estimate of design *c* when design *d* is detached is

$${\hat{\theta }}_{AC(d)}^{\text{net}}={\hat{\theta }}_{AC}^{\text{dir}}.$$

In this setting, $Q_{AC(d)}^{\text{inc}}=0$. Therefore, (5) can be rewritten as

(6) $$Q_{AC,d}^{\text{diff}}=\frac{1}{s_{AC}^{2}}{\left({\hat{\theta }}_{AC}^{\text{dir}}-{\hat{\theta }}_{AC}^{\text{net}}\right)}^{2}=\frac{1}{s_{AC}^{2}}{\left[\frac{s_{AC}^{2}}{s_{AC}^{2}+s_{AB}^{2}+s_{BC}^{2}}\left({\hat{\theta }}_{AC}^{\text{dir}}-{\hat{\theta }}_{AC}^{\text{ind}}\right)\right]}^{2}.$$

When *d*=*c*, the network estimate for design *c*, when the direct evidence for design *c* is excluded, is equal to the indirect evidence for design *c*

$${\hat{\theta }}_{AC(c)}^{\text{net}}={\hat{\theta }}_{AC}^{\text{ind}}.$$

Therefore, $Q_{c,c}^{\text{diff}}$ is calculated as

(7) $$Q_{AC,AC}^{\text{diff}}=Q_{AC}^{\text{inc}}-Q_{AC(AC)}^{\text{inc}}=\frac{1}{s_{AC}^{2}}\left[{\left({\hat{\theta }}_{AC}^{\text{dir}}-{\hat{\theta }}_{AC}^{\text{ind}}\right)}^{2}\left({\left(\frac{s_{AC}^{2}}{s_{AC}^{2}+s_{AB}^{2}+s_{BC}^{2}}\right)}^{2}-1\right)\right].$$

In both cases, (6) and (7) are scaled and squared versions of the inconsistency parameter (1). Thus, the net heat statistics are correlated with the formal inconsistency test statistic in this setting, in this example. However, these scaled versions of the inconsistency parameter have scaled chi‐squared distributions, making them awkward to interpret; why scale when the unscaled version has a known distribution?

### 4.2 Generalizing the net heat plot to a network with k+2 treatments where direct evidence is limited to specific comparisons

In this section, we use a more general network to illustrate the mathematics behind the net heat plot. We assume a network of two‐arm trials consisting of treatments *A* and *B* and additional treatments *X*_1_,*X*_2_,…,*X*_*k*_. In this network, there is only direct evidence comparing *A* versus *B*, *A* versus *X*_1_,*X*_2_,…,*X*_*k*_ and *B* versus *X*_1_,*X*_2_,…,*X*_*k*_. There are no trials directly comparing *X*_*i*_ and *X*_*j*_. We make the same assumptions as before: each trial has the same number of patients and each comparison has the same number of trials. Here, for simplicity, we assume the variance of the treatment effect, *s* ^2^, is common to all designs. We assume an equal weight of $\frac{1}{s^{2}}$ for each of the direct comparisons in the network so that each indirect comparison has weight $\frac{1}{2s^{2}}$. We let *c* be the design of interest (eg, *A* versus *B*), with direct estimate ${\hat{\theta }}_{c}^{\text{dir}}$. There are *k* possible indirect pathways, each involving a single additional node. Each additional node adds one loop to the network. Therefore, there are a total of *k*+2 treatments relevant to design *c*. Denote the indirect estimates by ${\hat{\theta }}_{c}^{\text{ind}(i)},i=1,\text{…},k$. The network estimate of *c* is equal to the weighted average of all the direct and indirect evidence combined, that is,

$${\hat{\theta }}_{c}^{\text{net}}=\frac{1}{k+2}\left\{2{\hat{\theta }}_{c}^{\text{dir}}+\overset{k}{\underset{i=1}{\sum }}{\hat{\theta }}_{c}^{\text{ind}(i)}\right\}.$$

To test the effect of detaching design *d*, there are two scenarios: *d*≠*c* and *d*=*c*. Assume first that *d*≠*c* and let the effect size for design *d* be ${\hat{\theta }}_{c}^{\text{ind}(d)}$. Then, when design *d* is detached, the remaining network evidence on *c* is

$${\hat{\theta }}_{c(d)}^{\text{net}}=\frac{1}{k+1}\left\{2{\hat{\theta }}_{c}^{\text{dir}}+\underset{i,i\neq d}{\sum }{\hat{\theta }}_{c}^{\text{ind}(i)}\right\}.$$

If, instead, the direct comparison, *d*=*c*, is detached, the network evidence remaining for design *c* is

$${\hat{\theta }}_{c(c)}^{\text{net}}=\frac{1}{k}\overset{k}{\underset{i=1}{\sum }}{\hat{\theta }}_{c}^{\text{ind}(i)}.$$

We now define ${\hat{\theta }}_{c(d/2)}^{\text{net}}$ as the average of all the network evidence for design *c* and the network evidence that remains for design *c* when design *d* is excluded so that

$${\hat{\theta }}_{c(d/2)}^{\text{net}}=\frac{1}{2}\left({\hat{\theta }}_{c(d)}^{\text{net}}+{\hat{\theta }}_{c}^{\text{net}}\right).$$

We write the difference between the network evidence on *c* when *d* is excluded and the network evidence on *c* in terms of ${\hat{\theta }}_{c}^{\text{ind}(i)}$ and putting it all together

(8) $$\begin{array}{ll} Q_{c,d}^{\text{diff}}=\; & \frac{2}{s^{2}}\left({\hat{\theta }}_{c(d)}^{\text{net}}-{\hat{\theta }}_{c}^{\text{net}}\right)\left[{\hat{\theta }}_{c}^{\text{dir}}-\frac{1}{2}\left({\hat{\theta }}_{c(d)}^{\text{net}}+{\hat{\theta }}_{c}^{\text{net}}\right)\right]\\ =\; & \frac{1}{s^{2}}\times \frac{1}{k+2}\left\{\frac{1}{k+1}\left(2{\hat{\theta }}_{c}^{\text{dir}}+\underset{i,i\neq d}{\sum }{\hat{\theta }}_{c}^{\text{ind}(i)}\right)-{\hat{\theta }}_{c}^{\text{ind}(d)}\right\}\\ & \times \left[2{\hat{\theta }}_{c}^{\text{dir}}\left(1-\frac{2k+3}{\left(k+1)(k+2\right)}\right)-\frac{1}{k+2}\left(\frac{2k+3}{k+1}\underset{i,i\neq d}{\sum }{\hat{\theta }}_{c}^{\text{ind}(i)}+{\hat{\theta }}_{c}^{\text{ind}(d)}\right)\right]. \end{array}$$

Else, if the direct comparison is detached,

$$Q_{c,c}^{\text{diff}}=-\frac{1}{s^{2}}\times \frac{4(k+1)}{{\left(k+2\right)}^{2}}{\left({\hat{\theta }}_{c}^{\text{dir}}-\frac{1}{k}\overset{k}{\underset{i=1}{\sum }}{\hat{\theta }}_{c}^{\text{ind}(i)}\right)}^{2}.$$

For *k*=1, the three‐treatment case, we obtain (6) and (7).

Suppose *k* is large so that *k*+1≈*k*; then, we can approximate (8) by

(9) $$\begin{array}{ll} Q_{c,d}^{\text{diff}} & \approx \frac{1}{s^{2}}\left\{\frac{1}{k}\left(2{\hat{\theta }}_{c}^{\text{dir}}+\underset{i,i\neq d}{\sum }{\hat{\theta }}_{c}^{\text{ind}(i)}\right)-{\hat{\theta }}_{c}^{\text{ind}(d)}\right\}\\ & \times \left[\frac{1}{k}\left\{2{\hat{\theta }}_{c}^{\text{dir}}-\frac{2}{k}\underset{i,i\neq d}{\sum }{\hat{\theta }}_{c}^{\text{ind}(i)}-\frac{1}{k}{\hat{\theta }}_{c}^{\text{ind}(d)}\right\}\right]. \end{array}$$

Essentially, (9) is a scaled product of two terms

$$Q_{c,d}^{\text{diff}}\approx \frac{1}{s^{2}}P_{1}P_{2},$$

where

$$P_{1}\approx \frac{1}{k}\left(2{\hat{\theta }}_{c}^{\text{dir}}+\underset{i,i\neq d}{\sum }{\hat{\theta }}_{c}^{\text{ind}(i)}\right)-{\hat{\theta }}_{c}^{\text{ind}(d)}$$

and

$$P_{2}\approx \frac{1}{k}\left\{\left({\hat{\theta }}_{c}^{\text{dir}}-{\hat{\theta }}_{c}^{\text{ind}}\right)+\left({\hat{\theta }}_{c}^{\text{dir}}-\frac{1}{k}\underset{i,i\neq d}{\sum }{\hat{\theta }}_{c}^{\text{ind}(i)}\right)\right\}.$$

Let ${\hat{\theta }}_{c}^{\text{ind}(-d)}=\text{average}\left(\sum_{i,i\neq d}{\hat{\theta }}_{c}^{\text{ind}(i)}\right)$; then, if *k* is large, we can simplify further

$$P_{1}\approx {\hat{\theta }}_{c}^{\text{ind}(-d)}-{\hat{\theta }}_{c}^{\text{ind}(d)}$$

$$P_{2}\approx \frac{2}{k}\left({\hat{\theta }}_{c}^{\text{dir}}-{\hat{\theta }}_{c}^{\text{ind}}\right).$$

Full details can be found in Appendix B (supplementary material).

Term *P*_1_ is the difference between the average indirect estimate for design *c* excluding design *d* and the indirect evidence for design *c* “from design *d*.” While the square of this is a plausible measure of the difference between the evidence coming from the loop including design *d* and the rest of the network (excluding the direct evidence), it is not specific to design *d* but to the loop including design *d*.

Term *P*_2_ is a scaled difference between the direct evidence for design *c* and the indirect evidence for design *c*. Term *P*_2_ can be large if the direct and indirect evidence differ and small if the direct and indirect evidence are similar. Therefore, in some cases, it could be a poor choice of multiplier for term *P*_1_.

We conclude that the terms used in the net heat plot neither generally identify designs causing inconsistency nor are necessarily relatively large if inconsistency is present (as *P*_2_ may be small).

## 5 SIMULATION STUDY: WHAT HAPPENS AS WE INCREASE THE NUMBER OF TREATMENT LOOPS IN A NETWORK?

In Section 4.2, we used equal variances to simplify calculations. However, this is unlikely to be realistic in most NMA cases. We now address this by using simulation to investigate what happens when we have the situation described in Section 4.2 where *P*_1_ is large, *P*_2_ is small, and we have unequal variances: our aim is to demonstrate that *P*_2_ is a poor choice of multiplier for *P*_1_. In more detail, the aim of this simulation study is to show, in a network in which we know there is inconsistency, that as the network increases in size, the ability of the net heat approach to identify this inconsistency is diminished.

We consider a network consisting of one treatment loop in which all the treatment effects are the same. We then inflate the treatment effect in one design to introduce inconsistency into the network. Treatment loops are added one at a time to the network and the values of $Q_{c,d}^{\text{diff}}$, $Q_{c}^{\text{inc}}$, and $Q_{c(d)}^{\text{inc}}$ are monitored. As above, $Q_{c}^{\text{inc}}$ quantifies the total amount of inconsistency for design *c* before detachment of design *d*. $Q_{c(d)}^{\text{inc}}$ quantifies the total amount of inconsistency for design *c* after detachment of design *d*. $Q_{c,d}^{\text{diff}}$ quantifies the reduction in inconsistency for design *c* following the detachment of design *d*.

Specifically, we start with a network consisting of one treatment loop (A,B,C). For each design, we simulate six trials. We generate the true treatment effects for each trial from designs AB and BC from a normal distribution with mean 0 and standard deviation 0.2. We generate the true treatment effect for the design AC for each trial from a normal distribution with mean 2 and standard deviation 0.2. This has the effect of introducing inconsistency between the direct and indirect evidence for the AC comparison. For each simulated trial treatment estimate, a corresponding standard error estimate is simulated from the normal distribution for the treatment effect with mean 0 and standard deviation 1. This ensures the standard error estimates are positive. As we move through the sequence of networks, each time we resimulate, the true treatment effects from these distributions. We repeat this process, adding one treatment at a time. At each stage, we have a network of two‐arm trials consisting of treatments *A* and *B* and additional treatments *X*_1_,*X*_2_,…,*X*_*k*_. There is only direct evidence comparing *A* versus *B*, *A* versus *X*_1_,*X*_2_,…,*X*_*k*_ and *B* versus *X*_1_,*X*_2_,…,*X*_*k*_. There are no trials directly comparing *X*_*i*_ and *X*_*j*_. We stopped when we reached 10 treatment loops. $Q_{c,d}^{\text{diff}}$, $Q_{c}^{\text{inc}}$, and $Q_{c(d)}^{\text{inc}}$ are calculated with *c*=AB and *d*=AC. R code can be found in Appendix D (supplementary material).

**Figure 4:**
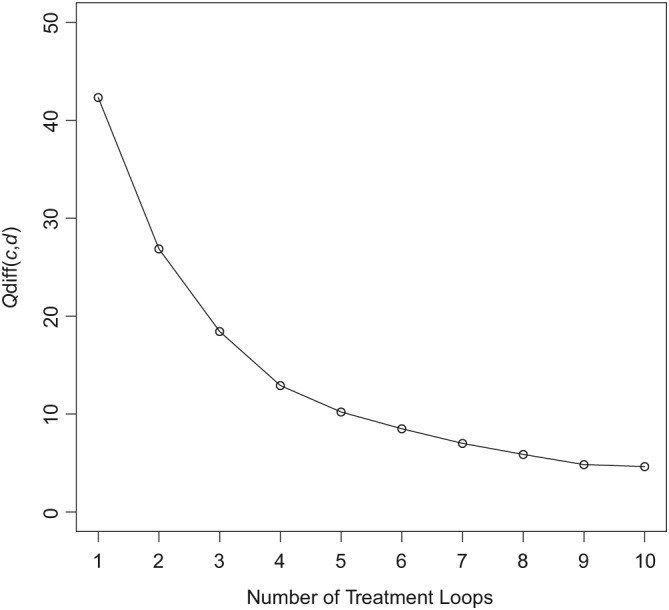
Plot showing the reduction in $Q_{c,d}^{\text{diff}}$ as the number of treatment loops in a network increases

In this situation, we know that before detachment of designs, inconsistency will be present between the direct and indirect estimates for the design AB because the indirect estimate for AB includes the inflated estimate of AC. Detaching design AC will then remove the inconsistency in the network, which will be quantified by $Q_{c,d}^{\text{diff}}$. Figure 4 plots $Q_{c,d}^{\text{diff}}$ against the number of treatment loops in the network. Estimates of $Q_{c,d}^{\text{diff}}$, $Q_{c}^{\text{inc}}$, and $Q_{c(d)}^{\text{inc}}$ are presented in Table S1 (supplementary material).

In terms of the notation used in Section 4.2, we expect to see that as we increase the number of treatment loops in the network, *P*_1_ remains the same, but *P*_2_ is reduced because adding more indirect evidence to the calculation of $\theta_{c}^{\text{ind}}$ “waters down” the direct evidence coming from design *d* and thus masking the inconsistency in the network, which shows that *P*_2_ is a poor choice of multiplier for *P*_1_.

Figure 4 and Table S1 (supplementary material) confirm this, showing that inconsistency due to design *d* in the net heat plot diminishes as the number of treatment loops increases but the amount of inconsistency in loop ABC remains the same. Therefore, as we increase the size of the network, the effect of inconsistency in one design is reduced so that in a network with a large number of loops, inconsistency will be hidden, ie, as we increase the amount of direct evidence on design *c*, the inconsistency in design *d* is masked. The net heat plot highlights concerns about inconsistency in a network when $Q_{c,d}^{\text{diff}}>8$. In this example, concerns about inconsistency are masked once there are seven or more treatment loops.

Inconsistency is a property of loops and as such the loop‐specific approaches considered in Sections 3.1, 3.2 and 3.3 are not affected by increasing the number of treatment loops in a network. However, node‐splitting models which compare the direct and indirect evidence for a comparison may be affected by increasing the number of consistent treatment loops. Therefore, we applied the node‐splitting approach to the same 10 simulated datasets. As expected, increasing the number of consistent treatment loops in the network (*ABX*_1_,*ABX*_2_,…) increased the sources of indirect evidence and reduced the effect of the indirect evidence from the ABC loop. The key differences between the net heat plot and the node‐splitting approach are that (1) the net heat plot multiplies *P*_1_ and *P*_2_ while claiming to identify when *P*_1_ is large (irrespective of *P*_2_) and (2) the node‐splitting approach gives a statistically valid estimate of *P*_2_ and test of the null hypothesis that it is zero.

## 6 APPLICATION OF METHODS FOR ASSESSING INCONSISTENCY

In this section, we apply the five methods for assessing inconsistency described in Section 3 to the lung cancer and diabetes networks.

### 6.1 Lung cancer network

We now apply the methods described in Section 3 to the lung cancer network introduced in Section 2.1. Cochran's Q statistic showed evidence of statistically significant heterogeneity in the whole network (*Q*=56.59, 40 df, p=0.043) and inconsistency between designs (*Q* ^inc^=4.52, 1 df, p=0.034. Heterogeneity within designs was close to the threshold of 0.05 but did not reach statistical significance (*Q* ^het^=52.07, 39 df, p=0.079). In the lung cancer network where there are no multi‐arm trials the loop inconsistency approach and Cochran's Q statistic are algebraically equivalent and therefore provide the same level of evidence for inconsistency in the lung cancer network. Letting A = RT, B = Seq CT, and C = Con CT, we have

$${\hat{\theta }}_{AB}^{\text{dir}}=-0.132,\mathrm{Var}\left({\hat{\theta }}_{AB}^{\text{dir}}\right)=0.036^{2}$$

$${\hat{\theta }}_{AC}^{\text{dir}}=-0.138,\mathrm{Var}\left({\hat{\theta }}_{AC}^{\text{dir}}\right)=0.039^{2}$$

$${\hat{\theta }}_{BC}^{\text{dir}}=-0.179,\mathrm{Var}\left({\hat{\theta }}_{BC}^{\text{dir}}\right)=0.062^{2}$$

$${\hat{\theta }}_{AC}^{\text{ind}}=-0.132+(-0.179)=-0.311,\mathrm{Var}\left({\hat{\theta }}_{AC}^{\text{ind}}\right)=0.036^{2}+0.062^{2}=0.072^{2}$$

$${\hat{\omega }}_{AC}=-0.138-(-0.311)=0.173,\mathrm{Var}({\hat{\omega }}_{AC})=0.039^{2}+0.036^{2}+0.062^{2}=0.082^{2}$$

$$z_{AC}=\frac{0.173}{0.082}=2.11,p=0.035.$$

To assess inconsistency and estimate both the direct and indirect evidence simultaneously, we conducted a NMA using the Royston‐Parmar time‐to‐event model, including a fixed effect inconsistency parameter following the method of Lu and Ades.^10^ The inconsistency parameter was fitted with a noninformative normal prior distribution. The inconsistency parameter was estimated as −0.176 (95% Credible Interval: −0.337, −0.016), giving an approximate p‐value of 0.032 and suggesting evidence of network inconsistency. Node‐splitting also resulted in p=0.033 for the difference between the direct and indirect evidence for each treatment comparison (Table 1).

**Table 1:** Node‐splitting results for the lung cancer network

	Direct		Indirect		Difference		
Comparison	Coef.	Std. Err.	Coef.	Std. Err.	Cef.	Std. Err.	P‐value
RT v Seq CT	0.131	0.036	0.043	0.074	−0.175	0.082	0.033
RT v Con CT	0.134	0.40	−0.309	0.072	0.175	0.082	0.033
Seq CT v Con CT	−0.177	0.063	−0.002	0.054	−0.175	0.082	0.033

Abbreviations: Con CT, radiotherapy plus concomitant chemotherapy; RT, radiotherapy; Seq CT, radiotherapy plus sequential chemotherapy.

**Figure 5:**
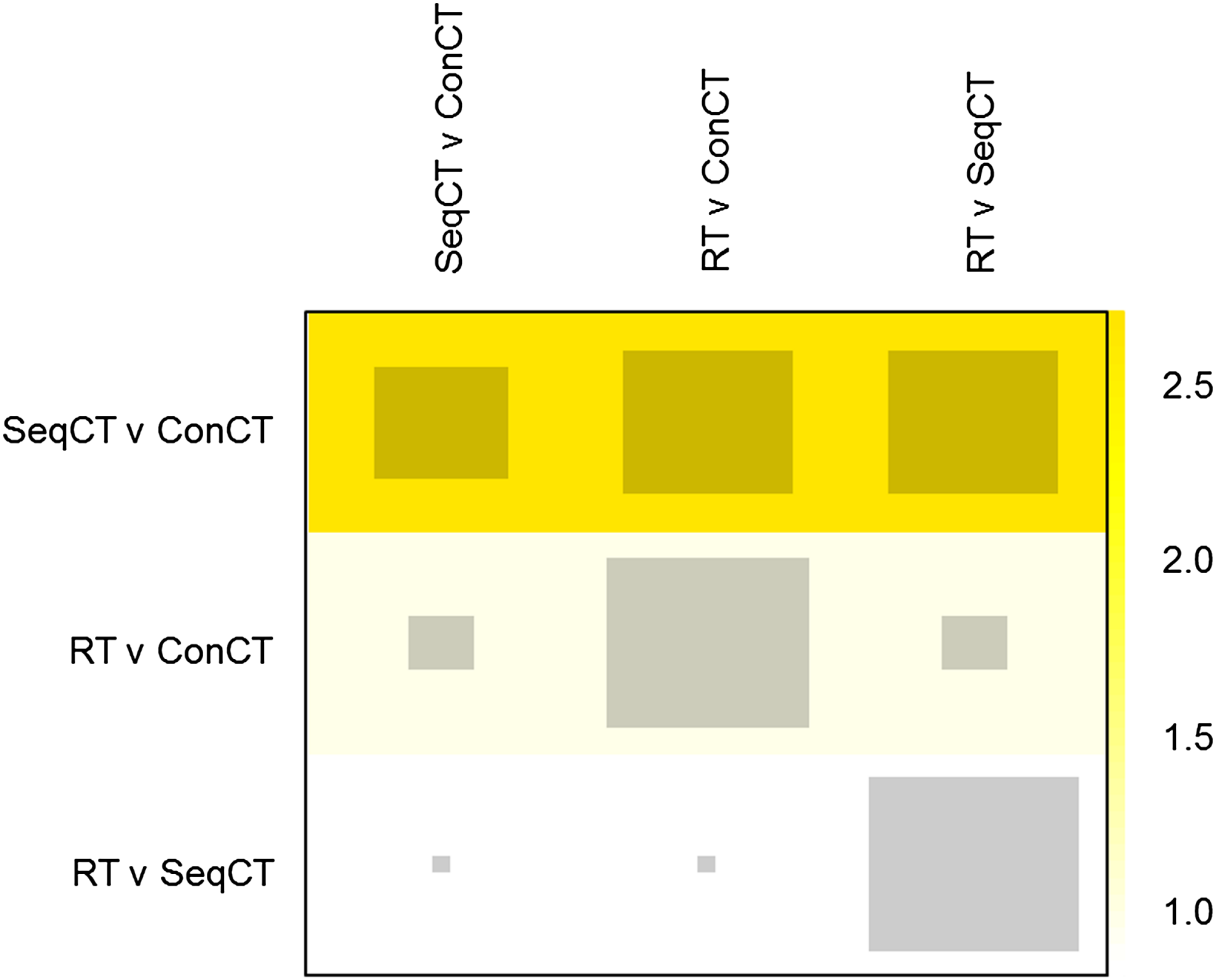
Net heat plot for the lung cancer network. Key to treatments: RT, radiotherapy; SeqCT, sequential chemotherapy; ConCT, concomitant chemotherapy [Colour figure can be viewed at http://wileyonlinelibrary.com]

The net heat plot is presented in Figure 5. The yellow colors indicate $Q_{c,d}^{\text{diff}}>0$. However, there are no areas of vibrant red, so it may be reasonable to conclude that there is no meaningful inconsistency in the lung cancer network, in contrast to the methods above. The difference in the shades of yellow suggests that inconsistency is most important in the Seq CT v Con CT treatment comparison. However, the Seq CT v Con CT comparison has the least amount of direct evidence, and therefore, the decomposition of *Q* has attributed the inconsistency mainly to this comparison.

To explore (6) further, we now calculate $Q_{c,d}^{\text{diff}}$. If we let *c* = AC be the comparison of interest, then ${\hat{\theta }}_{AB}^{\text{dir}}$, ${\hat{\theta }}_{AC}^{\text{dir}}$, ${\hat{\theta }}_{BC}^{\text{dir}}$, and ${\hat{\theta }}_{AC}^{\text{ind}}$ are as defined in Section 3.2. The network evidence for AC can be calculated as follows:

$${\hat{\theta }}_{AC}^{\text{net}}=\frac{0.036^{2}+0.062^{2}}{0.039^{2}+0.026^{2}+0.062^{2}}(-0.138)+\frac{0.039^{2}}{0.039^{2}+0.036^{2}+0.062^{2}}(-0.132+-0.179)=-0.1776$$

The Q statistics can be calculated from (3), (4), and (5) as follows:

$$Q_{AC}^{\text{inc}}=\frac{1}{s_{AC}^{2}}{\left({\hat{\theta }}_{AC}^{\text{dir}}-{\hat{\theta }}_{AC}^{\text{net}}\right)}^{2}=\frac{1}{0.039^{2}}{\left(-0.138--0.1776\right)}^{2}=1.026$$

$$\;Q_{AC(d)}^{\text{inc}}=\frac{1}{s_{AC}^{2}}{\left({\hat{\theta }}_{AC}^{\text{dir}}-{\hat{\theta }}_{AC(d)}^{\text{net}}\right)}^{2}=\frac{1}{0.039^{2}}{\left(-0.138--0.138\right)}^{2}=0$$

$$\;Q_{AC,d}^{\text{diff}}=Q_{AC}^{\text{inc}}-Q_{AC(d)}^{\text{inc}}=1.026,$$

which gives the same result as (6), indicating negligible inconsistency, in contrast, to a formal statistical test which rejects the null hypothesis with p=0.03.

### 6.2 Diabetes network

**Figure 6:**
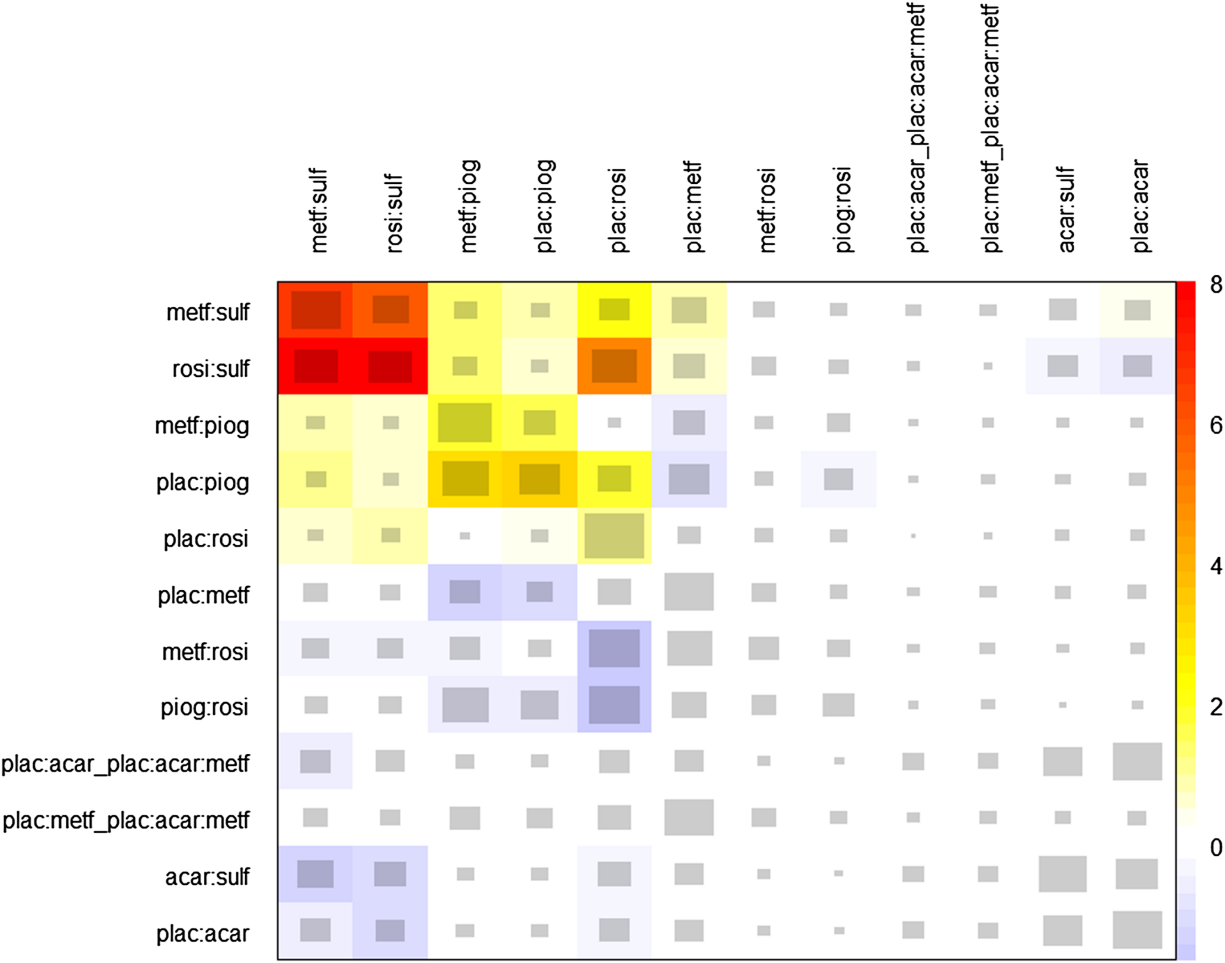
Net heat plot for the diabetes network. Key to treatments: acar, acarbose; benf, benfluorex; metf, metformin; migl, miglitol; piog, pioglitazone; plac, placebo; rosi, rosiglitazone; sita, sitagliptin; sulf, sulfonylurea; vild, vildagliptin [Colour figure can be viewed at http://wileyonlinelibrary.com]

We now apply the methods described in Section 3 to the diabetes network introduced in Section 2.2. Cochran's Q statistic showed evidence of statistically significant inconsistency between designs (*Q* ^inc^=22.53, 7df, p=0.002) and within designs (*Q* ^het^=74.46, 11df, p<0.001). The net heat plot (Figure 6) raises concerns about inconsistency ( $Q_{c,d}^{\text{diff}}>8$) within the metformin (metf), sulfonylurea (sulf), and rosiglitazone (rosi) treatment loop and particularly the comparisons involving sulfonylurea. However, the loop inconsistency and node‐splitting approaches are able to formally test this. Letting A = metformin, B = sulfonylurea, and C = rosiglitazone, for the diabetes network and following the loop inconsistency approach outlined in Section 3.2, we have

$${\hat{\theta }}_{AB}^{\text{dir}}=-0.370,\mathrm{Var}\left({\hat{\theta }}_{AB}^{\text{dir}}\right)=0.014$$

$$\;{\hat{\theta }}_{AC}^{\text{dir}}=0.073,\mathrm{Var}\left({\hat{\theta }}_{AC}^{\text{dir}}\right)=0.026$$

$$\;{\hat{\theta }}_{BC}^{\text{dir}}=1.20,\mathrm{Var}\left({\hat{\theta }}_{BC}^{\text{dir}}\right)=0.021$$

$$\;{\hat{\theta }}_{BC}^{\text{ind}}=0.073-(-0.370)=0.443,\mathrm{Var}\left({\hat{\theta }}_{AC}^{\text{ind}}\right)=0.026+0.014=0.040$$

$${\hat{\omega }}_{BC}=1.20-0.443=0.757,\mathrm{Var}({\hat{\omega }}_{AC})=0.021+0.026+0.014=0.061$$

$$z_{BC}=\frac{0.757}{\sqrt{0.061}}=3.07,p=0.002.$$

The results of node‐splitting in the diabetes network are presented in Table S2 (supplementary material). For the sulfonylurea and rosiglitazone and sufonylurea and metformin comparisons, p<0.001, suggesting evidence of important inconsistency within the diabetes network.

We have not applied the inconsistency parameter approach to the diabetes network. In a large network such as the diabetes network, it is computationally simpler to use the node‐splitting approach instead.

In this example, the net heat plot is in agreement with the loop inconsistency and node‐splitting approaches with all three identifying important inconsistency within the metformin, sulfonylurea, and rosiglitazone treatment loop. All three approaches also identified the treatment loop metformin, pioglitazone (piog), and placebo (plac) as an area of concern. The net heat plot colors this treatment loop yellow ( $Q_{c,d}^{\text{diff}}\approx 4$), suggesting that although inconsistency may be present, it is not important. The loop inconsistency approach is able to formally test this and reaches a similar conclusion (z=1.80, p=0.073). The node‐splitting approach also suggests evidence of important inconsistency in the network (Table S2, supplementary material).

In this example, the net heat plot, the loop inconsistency approach, and node splitting all identified the same treatment loops as potential sources of inconsistency in the network. However, the loop inconsistency and node splitting approaches are able to formally test inconsistency in loops. Therefore, in this example, node‐splitting is advantageous over the net heat plot because it not only assesses all the treatment loops in the network but is also able to formally test for evidence of important inconsistency.

## 7 CONCEPTUAL CRITIQUE OF THE NET HEAT PLOT

The net heat plot aims to identify a specific design (or designs) that drive inconsistency in a network. However, locating inconsistency to a specific design (or even a pair of designs) is a difficult and sometimes impossible task since inconsistency arises from comparisons between at least three designs. In a three‐treatment network, inconsistency can only be identified and not actually located. Thus, any attempt to locate inconsistency within designs is potentially misleading, in particular because it may tend to attribute inconsistency to areas with less evidence. For example, in Figure 5, the difference in the shades of yellow suggests that inconsistency is most important in the Seq CT v Con CT treatment comparison. However, the Seq CT v Con CT comparison has the least amount of direct evidence, and therefore, the decomposition of *Q* has attributed the inconsistency mainly to this comparison. We expect something similar would also happen in more complex networks.

Within a network one (or more) deviating direct comparison(s) may affect the network estimates of other comparisons, producing hot spots of inconsistency, ie, treatment comparisons responsible for inconsistency in one or more treatment loops.^12^ The very concept of a “hot spot” is not clearly defined by Krahn et al,^12^ and the asymmetric nature of the net heat plot makes interpretation harder. In addition, Krahn et al^12^ were unclear about how the intensity of color in the net heat plot relates to important, clinically meaningful inconsistency. For example, in Figure 5, the yellow colors indicate $Q_{c,d}^{\text{diff}}>0$. However, for our lung cancer network, there are no areas of vibrant red, so it may be reasonable to conclude that there is no meaningful inconsistency in the lung cancer network, in contrast to Section 6.1.

Inconsistency is a loop property; it does not make sense at the level of an individual design. Further, it cannot be linked to a specific design in the loop unless at least one design is part of more than one loop. In other words, locating inconsistency within a network depends on the structure of the network, and no simple method works for all networks. Identifying inconsistency will depend to some extent on the network connectedness and the number of treatments and trial designs. Indeed, if more than one design deviates from the true effect, then it is possible that inconsistency might be masked. Similarly, inconsistency might be harder to spot in a fully connected network, where there are numerous pathways of indirect evidence, than in a network with fewer direct (and indirect) connections.

Unlike *Q*, *Q* ^het^, and *Q* ^inc^, which follow chi‐squared distributions, $Q_{c,d}^{\text{diff}}$ as the difference between two approximately chi‐square distributed, correlated components, has a nonstandard distribution and is therefore hard to interpret. Complex calculations would be required to calculate the sampling distribution and obtain a p‐value. One possibility would be to use bootstrapping, but since $Q_{c,d}^{\text{diff}}$ does not have a natural interpretation, we did not pursue this.

Ideally, what is needed is a way to combine the graphical approach utilized by the net heat plot with the results of the formal statistical tests implemented in the node‐splitting and loop inconsistency approaches to produce a graphically accessible way for identifying inconsistency in networks.

## 8 DISCUSSION

Inconsistency in a network can lead to biased treatment effect estimates; therefore, it is important that attempts are made to identify, understand, and adjust for inconsistency. There are many methods for assessing inconsistency in NMA. In this paper, we considered five of the most popular methods from the simplest method of loop inconsistency^9^ to more complex models such as the inconsistency parameter approach^10^ and the graphical net heat approach.^12^

The net heat plot calculates the change in inconsistency across the network caused by relaxing the consistency assumption for each design. The change in inconsistency is known as $Q_{c,d}^{\text{diff}}$, and these values are displayed graphically in the net heat plot. We derived a formula for $Q_{c,d}^{\text{diff}}$, which could be applied to a network in which two treatments are both directly compared with other treatments to quantify the amount of inconsistency in the network using the net heat plot. We have shown that $Q_{c,d}^{\text{diff}}$ can be difficult to interpret and, in some cases, a misleading measure of inconsistency. In the special case of three‐treatment networks, it is approximately an arbitrary scaled version of the difference between the direct and the indirect evidence, which explains why, in the lung cancer example, the net heat plot did not identify the same possibility of inconsistency as the analyses in Section 6.1. We advise that the net heat plot is interpreted with caution.

The net heat plot uses Cochran's Q statistic^8^ in a fixed effect framework and decomposes it into within‐trial and between‐trial heterogeneity. This reflects the fact that heterogeneity and inconsistency can be considered as different aspects of heterogeneity, where inconsistency is the discrepancy between results of single studies and predictions based on a consistency model.^12^ The within‐trial and between‐trial heterogeneity statistics are assumed to follow chi‐squared distributions. The lung cancer example showed little evidence of heterogeneity, and therefore, it was appropriate, for this example, to use a fixed effect model that assumed that there was no heterogeneity within designs. Although more complex, the calculations in Section 4.2 could be conducted using a random effects model, and this may be more appropriate when heterogeneity is present in a network. However, further investigation is required to determine how the net heat plot identifies inconsistency when heterogeneity is present.

In this paper, we have shown through simulation that inconsistency in larger networks may be hidden when using the net heat plot alone (Figure 4). We have also shown that the statistics on which the net heat plot is built are sensible in some scenarios but have a somewhat arbitrary weighting. In all scenarios, they are scaled versions of the loop inconsistency test statistic and as such have scaled chi‐squared distributions. However, as Hoaglin^27^ discusses, the Q statistics only approach the chi‐squared distribution if the study sizes are large (mainly because the standard errors are generally not known but estimates), which may not be the case in many meta‐analyses. While this can be important in applications, it does not invalidate our arguments in this paper. Therefore, in all situations, the statistics behind the net heat plot are unintuitive, awkward to interpret, and do not lend themselves to statistical testing. Furthermore, we have shown that the statistics underpinning the net heat plot can neither generally identify designs causing inconsistency nor are they necessarily relatively large if inconsistency is present. Hence, inconsistency in larger networks may be hidden when the net heat plot is used on its own to identify inconsistency. Therefore, it may be that no one method should be considered alone for assessing inconsistency and that a combination of approaches is the best way forward although this introduces the challenge of interpreting potentially conflicting results from multiple tests.

Throughout this paper, except for the diabetes network, we assumed all networks contained two‐arm trials only, and the indirect evidence for a design was assumed to come from pathways involving one additional treatment only. While this is unlikely to be true in larger networks, the weighting of the indirect evidence gets smaller as more additional treatments are involved so the contribution of longer pathways to the indirect evidence is minimal. Furthermore, we have shown that the net heat approach can be misleading when only considering two arm trials. Therefore, given the added complexity of including multiarm trials in a network, it is likely that interpreting the net heat plot will only become more problematic with increasing network complexity.

Using the loop inconsistency approach to test for inconsistency within each loop leads to problems with multiple testing and can be cumbersome in networks with many treatment loops. By contrast, the inconsistency parameter approach is straight forward to incorporate within most NMA models and quantifies inconsistency but does not provide a straight forward way for locating the inconsistency. In large networks, the net heat plot is straight forward to implement, and the provision of freely available user‐friendly software is likely to increase the popularity of the approach. Previously, node‐splitting was cumbersome in large networks as each comparison of interest requires a separate model. However, a decision rule that chooses which comparisons to split, only selecting comparisons in potentially inconsistent loops but ensuring that all potentially inconsistent loops in the network are investigated, has eliminated most of the manual work involved in using the node‐splitting approach, even in large networks.^28^ Furthermore, node‐splitting has the added advantage over the net heat approach of being able to statistically test for evidence of inconsistency.

Other methods of assessing inconsistency which have not been considered in this paper include the design‐by‐treatment interaction model,^5^, ^29^ random inconsistency effects,^30^, ^31^, ^32^, ^33^, ^34^ factorial analysis of variance,^35^ generalized linear mixed models,^36^, ^37^ and the two‐stage approach.^38^ Furthermore, if covariates are distributed unevenly between trials, then inconsistency may be reduced by adjusting for covariates.^39^, ^40^ For a review of methods for assessing inconsistency in NMA, we recommend Donegan et al.^15^ All methods to assess inconsistency should be interpreted cautiously, taking the clinical context into account.

In MA, forest plots can be used to check for outlying single studies and highly weighted studies, which can both be influential. In NMA where evidence for a treatment comparison comes from several sources, a forest plot may not provide all the information necessary for assessing influential trials or designs. Additional complexity arises when a network includes multiarm trials. Therefore, careful exploratory work plus presenting the results as in Figure 2 are the key rather than the net heat plot.^41^ Furthermore, recent work to reduce the cumbersome nature of using node‐splitting in large networks^28^ means that an accessible graphical display of node‐splitting results may be the graphical representation of inconsistency that analysts need to identify inconsistency in their NMAs.

It is important that attempts are made to identify, understand, and adjust for inconsistency in a network. The net heat plot is an arbitrary weighting of the loop inconsistency statistics, which does not lend itself to statistical testing and can mask inconsistency in larger networks. We advise that the net heat plot is used with caution. Alternative graphical methods to the net heat plot, which appropriately assess the amount of inconsistency within a network and display the results graphically, clearly highlighting influential and inconsistent designs, are needed.

## Supporting information

SIM_8383‐Supp‐0001‐Net heat supplementary material.pdf
