# Supplementary material for: Identifying inconsistency in network meta‐analysis: Is the net heat plot a reliable method?
Source: Stat Med. 2019 Oct 24;38(29):5547–64. doi: 10.1002/sim.8383 (PMC6899484; doi:10.1002/sim.8383)
Supplement: Supplementary file 1 — SIM_8383‐Supp‐0001‐Net heat supplementary material.pdf [file SIM-38-5547-s001.pdf]

Identifying inconsistency in network meta-analysis: Is the net heat plot a reliable method? Supplementary material

S. C. Freeman, D. Fisher, I. R. White, A. Auperin, J. R. Carpenter

A List of RCTs included in Lung Cancer network

Alberti W, Niederle N, Budach V, Konietzko N, Sack H. Prospective randomized study comparing immediate radiotherapy, chemo- plus radiotherapy or delayed radiotherapy in non-small cell lung cancer. Journal of Cancer Research and Clinical Oncology. 1990;116(Part I):503.

Anderson G, Deeley TJ, Smith C, Jani J. Comparison of radiotherapy alone and radiotherapy with chemotherapy using adriamycin and 5-fluorouracil in bronchogenic carcinoma. Thorax. 1981;36:190-3.

Atagi S, Kawahara M, Tamura T, Noda K, Watanabe K, Yokoyama A, et al. Standard thoracic radiotherapy with or without concurrent daily low-dose carboplatin in elderly patients with locally advanced non-small cell lung cancer: A phase III trial of the Japan Clinical Oncology Group (JCOG9812). Jpn J Clin Oncol. 2005;35(4):195-201.

Ball D, Bishop J, Smith J, O'Brien P, Davis S, Ryan G, et al. A randomised phase III study of accelerated or standard fraction radiotherapy with or without concurrent carboplatin in inoperable non-small cell lung cancer: Final report of an Australian multi-centre trial. Radiotherapy and Oncology. 1999;52:129-36.

Belani CP, Choy H, Bonomi P, Scott C, Travis P, Haluschak J, et al. Combined chemoradiotherapy regimens of paclitaxel and carboplatin for locally advanced non-small-cell lung cancer: A randomized phase II locally advanced multi-modality protocol. J Clin Oncol. 2005;23(25):5883-91.

Belderbos J, Uitterhoeve L, van Zandwijk N, Belderbos H, Rodrigus P, van de Vaart P, et al. Randomised trial of sequential versus concurrent chemo-radiotherapy in patients with inoperable non-small cell lung cancer (EORTC 08972-22973). Eur J Cancer. 2007;43(1):114-21.

Blanke C, Ansari R, Mantravadi R, Gonin R, Tokars R, Fisher W, et al. Phase III trial of thoracic irradiation with or without cisplatin for locally advanced unresectable non-small-cell lung cancer: A Hoosier Oncology Group Protocol. J Clin Oncol. 1995;13:1425-9.

Bonner JA, McGinnis WL, Stella PJ, Marschke Jr R, Sloan JA, Shaw EG, et al. The possible advantage of hyperfractionated thoracic radiotherapy in the treatment of locally advanced nonsmall cell lung

carcinoma. Cancer. 1998;82:1037-48.

Brodin O, Nou E, Mercke C, Linden C, Lundstrom R, Arwidi A, et al. Comparison of induction chemotherapy before radiotherapy with radiotherapy only in patients with locally advanced squamous cell carcinoma of the lung. Eur J Cancer. 1996;32A(11):1893-900.

Cardiello C, Blanco Villalba J, Anac S, Francheri Wilson C, Trodler C, Hunis A, et al. Combined radiochemotherapy (RTCT) versus radiotherapy (RT) in limited inoperable non small cell carcinoma of the lung (NSCLC). Proceedings American Society of Clinical Oncology. 1985;4:177.

Clamon G, Herndon J, Cooper R, Chang AY, Rosenman J, Green MR. Radiosensitization With Carboplatin for Patients With Unresectable Stage III NonSmall-Cell Lung Cancer: A Phase III Trial of the Cancer and Leukemia Group B and the Eastern Cooperative Oncology Group. J Clin Oncol. 1999;17:4-11.

Clamon G, Herndon J, Eaton W, Rosenman J, Maurer L, Cooper M, et al. A feasibility study of extended chemotherapy for locally advanced non-small cell lung cancer: A phase II trial of Cancer and Leukemia Group B. Cancer Invest. 1994;12:273-82.

Crino L, Latini P, Meacci M, Corgna E, Maranzano E, Darwish S, et al. Induction chemotherapy plus high-dose radiotherapy versus radiotherapy alone in locally advanced unresectable non-small-cell lung cancer. Annals of Oncology. 1993;4:847-51.

Cullen M, Billingham L, Woodroffe C, Chetiyawardana A, Gower N, Joshi R, et al. Mitomycin, Ifosfamide, and Cisplatin in Unresectable NonSmall-Cell Lung Cancer: Effects on Survival and Quality of Life. J Clin Oncol. 1999;17:3188-94.

Cuneyt Ulutin H, Pak Y. Preliminary results of radiotherapy with or without weekly paclitaxel in locally advanced non-small cell lung cancer. J Cancer Res Clin Oncol. 2003;129(1):52-6.

Curran WJ, Jr., Paulus R, Langer CJ, Komaki R, Lee JS, Hauser S, et al. Sequential vs. concurrent chemoradiation for stage III non-small cell lung cancer: Randomized phase III trial RTOG 9410. J Natl Cancer Inst. 2011;103(19):1452-60.

Dillman R, Seagren S, Propert K, Guerra J, Eaton W, Perry M, et al. A randomized trial of induction

chemotherapy plus high-dose radiation versus radiation alone in stage III non-small cell lung cancer. N Engl J Med. 1990;323:940-5.

Douillard J, Gervais R, Quoix E, Chevalier T, Groumellec A, Lemarie E, et al. Randomized phase III trial for stage III unresectable non small cell lung cancer: Induction chemotherapy [vinorelbine-cisplatin] followed by conventional radiation without or with daily carboplatin. Lung Cancer. 2005;49 (suupl 2):S16 (abstract O-040).

Fairlamb D, Milroy R, Gower N, Parmar M, Peake M, Rudd R, et al. A randomised comparison of radical radiotherapy with or without chemotherapy for patients with non-small cell lung cancer: Results from the Big Lung Trial. Radiotherapy and Oncology. 2005;75(2):134-40.

Fournel P, Robinet G, Thomas P, Souquet PJ, Lena H, Vergnenegre A, et al. Randomized phase III trial of sequential chemoradiotherapy compared with concurrent chemoradiotherapy in locally advanced non-small-cell lung cancer: Groupe Lyon-Saint-Etienne d'Oncologie Thoracique-Groupe Francais de Pneumo-Cancerologie NPC 95-01 Study. J Clin Oncol. 2005;23(25):5910-7.

Furuse K, Fukuoka M, Kawahara M, Nishikawa H, Takada Y, Kudoh S, et al. Phase III study of concurrent versus sequential thoracic radiotherapy in combination with mitomycin, vindesine, and cisplatin in unresectable stage III nonsmall-cell lung cancer. J Clin Oncol. 1999;17:2692-9.

Gregor A, Macebth FR, Paul J, Cram L, Hansen H. Radical radiotherapy and chemotherapy in localized inoperable non-small-cell lung cancer: A randomized trial. J Natl Cancer Inst. 1993;85(12):997.

Groen HJM. Continuously infused carboplatin used as radiosensitizer in locally unresectable non-small-cell lung cancer: A multicenter phase III study. Annals of Oncology. 2004;15(3):427-32.

Huber RM, Flentje M, Schmidt M, Pollinger B, Gosse H, Willner J, et al. Simultaneous chemoradiotherapy compared with radiotherapy alone after induction chemotherapy in inoperable stage IIIA or IIIB non-small-cell lung cancer: Study CTRT99/97 by the Bronchial Carcinoma Therapy Group. J Clin Oncol. 2006;24(27):4397-404.

Jeremic B, Shibamoto Y, Acimovic L, Djuric L. Randomized trial of hyperfractionated radiation therapy with or without concurrent chemotherapy for stage III non-small-cell lung cancer. J Clin Oncol.

1995;13:452-8.

Jeremic B, Shibamoto Y, Acimovic L, Milisavljevic S. Hyperfractionated radiation therapy with or without concurrent low-dose daily carboplatin/etoposide for stage III non-small-cell lung cancer: A randomized study. J Clin Oncol. 1996;14:1065-70.

Le Chevalier T, Arrigada R, Quoix E, Ruffie P, Martin M, Tarayre M, et al. Radiotherapy alone versus combined chemotherapy and radiotherapy in nonresectable non-small cell lung cancer: First analysis of a randomized trial in 353 patients. J Natl Cancer Inst. 1991;83:417-23.

Mattson K, Holsti LR, Holsti P, Jakobsson M, Kajanti M, Liippo K, et al. Inoperable non-small cell lung cancer: Radiation with or without chemotherapy. Eur J Cancer Clin Oncol. 1988;24(3):477-82.

Mattson KV. Docetaxel as neoadjuvant therapy for radically treatable stage III non-small-cell lung cancer: A multinational randomised phase III study. Annals of Oncology. 2003;14(1):116-22.

Mira JG, Miller TP, Crowley JJ, editors. Chest irradiation (RT) vs chest RT + chemotherapy prophylactic brain RT in localized non small cell lung cancer: A Southwest Oncology Group randomized study. 32nd Annual ASTRO Meeting; 1990.

Morton R, Jett J, McGinnis WL, Earle J, Therneau T, Krook J, et al. Thoracic radiation therapy alone compared with combined chemoradiotherapy for locally unresectable non-small cell lung cancer. A randomized phase III trial. Ann Intern Med. 1991;115:681-6.

Planting A, Helle P, Drings P, Dalesio O, Kirkpatrick A, McVie G, et al. A randomized study of high-dose split course radiotherapy preceded by high-dose chemotherapy versus high-dose radiotherapy only in locally advanced non-small-cell lung cancer. Annals of Oncology. 1996;7:139-44.

Sarihan S, Kayisogullari U, Ercan I, Engin K. Randomized phase II study of radiotherapy alone versus radiotherapy with paclitaxel in non-small cell lung cancer. The Journal of International Medical Research. 2004;32:375-83.

Sause W, Kolesar P, Taylor IV S, Johnson D, Livingston R, Komaki R, et al. Final results of phase III trial in regionally advanced unresectable non-small cell lung cancer. Chest. 2000;117:358-64.

Scagliotti GV, Szczesna A, Ramlau R, Cardenal F, Mattson K, Van Zandwijk N, et al. Docetaxel-based induction therapy prior to radiotherapy with or without docetaxel for non-small-cell lung cancer. Br J Cancer. 2006;94(10):1375-82.

Schaake-Koning C, van den Bogaert W, Dalesio O, Festen J, Hoogenhout J, van Houtte P, et al. Effects of concomitant cisplatin and radiotherapy on inoperable non-small cell lung cancer. N Engl J Med. 1992;326:524-30.

Trovo MG, Minatel E, Franchin G, Boccieri MG, Nascimben O, Bolzicco G, et al. Radiotherapy versus radiotherapy enhanced by cisplatin in stage III non-small cell lung cancer. Int J Radiation Oncology Biology Physics. 1992;24:11-5.

van Houtte P, Klastersky J, Renaud A, Michel J, Vandermoten G, Nguyen H, et al. Induction chemotherapy with cisplatin, etoposide and vindesine before radiation therapy with non-small cell lung cancer. Antibiot Chemother. 1988;41.

White J, Chen T, Reed R, Mira JG, Stuckey W, Weatherall Y, et al. Limited squamous cell carcinoma of the lung: A Southwest Oncology Group randomized study of radiation with or without doxorubicin chemotherapy and with or without levamisole immunotherapy. Cancer Treat Rep. 1982;66:1113-20.

B Statistical Details

This appendix provides further details on Section 4.2.

The network estimate of c is equal to the weighted average of all the direct and indirect evidence combined; that is:

$$\hat{\theta}_c^{\text{net}} = \frac{1}{k+2}\left\{2\hat{\theta}_c^{\text{dir}} + \sum_{i=1}^{k}\hat{\theta}_c^{\text{ind}(i)}\right\}.$$

Assume that $d \neq c$ and let the effect size for design d be $\hat{\theta}_c^{\text{ind}(d)}$. When design d is detached the remaining network evidence on c is:

$$\hat{\theta}_{c(d)}^{\text{net}} = \frac{1}{k+1}\left\{2\hat{\theta}_c^{\text{dir}} + \sum_{i,i\neq d}\hat{\theta}_c^{\text{ind}(i)}\right\}$$

If the direct comparison, $d = c$, is detached the network evidence remaining for design c is:

$$\hat{\theta}_{c(c)}^{\text{net}} = \frac{1}{k}\sum_{i=1}^{k}\hat{\theta}_c^{\text{ind}(i)}$$

When $d \neq c$, $\hat{\theta}_c^{\text{net}}$ can be re-written in terms of $\hat{\theta}_c^{\text{ind}(i)}$ as follows:

$$\hat{\theta}_c^{\text{net}} = \frac{1}{k+2}\left\{2\hat{\theta}_c^{\text{dir}} + \hat{\theta}_c^{\text{ind}(d)} + \sum_{i,i\neq d}\hat{\theta}_c^{\text{ind}(i)}\right\}.$$

The inconsistency Q statistics were defined in Section 4.1 as (3), (4) and (5). Expanding (5) and rearranging:

$$Q_{c,d}^{\text{diff}} = \frac{2}{s^2}\left(\hat{\theta}_{c(d)}^{\text{net}} - \hat{\theta}_c^{\text{net}}\right)\left[\hat{\theta}_c^{\text{dir}} - \frac{1}{2}\left(\hat{\theta}_{c(d)}^{\text{net}} + \hat{\theta}_c^{\text{net}}\right)\right]$$

Define $\hat{\theta}_{c(d/2)}^{\text{net}}$ as the average of all the network evidence for design c and the network evidence that remains for design c when design d is excluded so that:

$$\hat{\theta}_{c(d/2)}^{\text{net}} = \frac{1}{2}\left(\hat{\theta}_{c(d)}^{\text{net}} + \hat{\theta}_c^{\text{net}}\right)$$

Then we can write:

$$Q_{c,d}^{\text{diff}} = \frac{2}{s^2}\left(\hat{\theta}_{c(d)}^{\text{net}} - \hat{\theta}_c^{\text{net}}\right)\left(\hat{\theta}_c^{\text{dir}} - \hat{\theta}_{c(d/2)}^{\text{net}}\right)$$

Write the difference between the network evidence on c when d is excluded and the network evidence on c in terms of $\hat{\theta}_c^{\text{ind}(i)}$, as follows:

$$\hat{\theta}_{c(d)}^{\text{net}} - \hat{\theta}_c^{\text{net}} = \frac{1}{k+2}\left\{\frac{1}{k+1}\left(2\hat{\theta}_c^{\text{dir}} + \sum_{i,i\neq d}\hat{\theta}_c^{\text{ind}(i)}\right) - \hat{\theta}_c^{\text{ind}(d)}\right\}$$

and similarly

$$\hat{\theta}_{c(d/2)}^{\text{net}} = \frac{1}{2}\left(\hat{\theta}_{c(d)}^{\text{net}} + \hat{\theta}_c^{\text{net}}\right) = \frac{1}{2}\times\frac{1}{k+2}\left\{\frac{2k+3}{k+1}\left(2\hat{\theta}_c^{\text{dir}} + \sum_{i,i\neq d}\hat{\theta}_c^{\text{ind}(i)}\right) + \hat{\theta}_c^{\text{ind}(d)}\right\}$$

Putting it all together we get (8):

$$Q_{c,d}^{\text{diff}} = \frac{1}{s^2}\times\frac{1}{k+2}\left\{\frac{1}{k+1}\left(2\hat{\theta}_c^{\text{dir}} + \sum_{i,i\neq d}\hat{\theta}_c^{\text{ind}(i)}\right) - \hat{\theta}_c^{\text{ind}(d)}\right\}$$
$$\times\left[2\hat{\theta}_c^{\text{dir}}\left(1 - \frac{2k+3}{(k+1)(k+2)}\right) - \frac{1}{k+2}\left(\frac{2k+3}{k+1}\sum_{i,i\neq d}\hat{\theta}_c^{\text{ind}(i)} + \hat{\theta}_c^{\text{ind}(d)}\right)\right]$$

Or, if the direct comparison is detached:

$$Q_{c,c}^{\text{diff}} = -\frac{1}{s^2}\times\frac{4(k+1)}{(k+2)^2}\left(\hat{\theta}_c^{\text{dir}} - \frac{1}{k}\sum_{i=1}^{k}\hat{\theta}_c^{\text{ind}(i)}\right)^2$$

Suppose k is large so that $k+1 \approx k$ then we can approximate (8) by:

$$Q_{c,d}^{\text{diff}} \approx \frac{1}{s^2}\cdot\frac{1}{k}\left\{\frac{1}{k}\left(2\hat{\theta}_c^{\text{dir}} + \sum_{i,i\neq d}\hat{\theta}_c^{\text{ind}(i)}\right) - \hat{\theta}_c^{\text{ind}(d)}\right\}$$
$$\times\left[2\hat{\theta}_c^{\text{dir}}\left(1 - \frac{2}{k}\right) - \frac{1}{k}\left(2\sum_{i,i\neq d}\hat{\theta}_c^{\text{ind}(i)} + \hat{\theta}_c^{\text{ind}(d)}\right)\right].$$

Let $1 - \frac{2}{k} \approx 1$, then:

$$Q_{c,d}^{\text{diff}} \approx \frac{1}{s^2}\left\{\frac{1}{k}\left(2\hat{\theta}_c^{\text{dir}} + \sum_{i,i\neq d}\hat{\theta}_c^{\text{ind}(i)}\right) - \hat{\theta}_c^{\text{ind}(d)}\right\}$$
$$\times\left[\frac{1}{k}\left\{2\hat{\theta}_c^{\text{dir}} - \frac{2}{k}\sum_{i,i\neq d}\hat{\theta}_c^{\text{ind}(i)} - \frac{1}{k}\hat{\theta}_c^{\text{ind}(d)}\right\}\right]$$

Now $\hat{\theta}_c^{\text{ind}}$ is the average of all the indirect evidence across the whole network:

$$\hat{\theta}_c^{\text{ind}} = \frac{1}{k}\left(\sum_{i,i\neq d}\hat{\theta}_c^{\text{ind}(i)} + \hat{\theta}_c^{\text{ind}(d)}\right).$$

Then:

$$\begin{aligned}
Q_{c,d}^{\text{diff}} &\approx \frac{1}{s^2}\left\{\frac{1}{k}\left(2\hat{\theta}_c^{\text{dir}} + \sum_{i,i\neq d}\hat{\theta}_c^{\text{ind}(i)}\right) - \hat{\theta}_c^{\text{ind}(d)}\right\} \\
&\quad \times \left[\frac{1}{k}\left\{2\hat{\theta}_c^{\text{dir}} - \frac{1}{k}\sum_{i,i\neq d}\hat{\theta}_c^{\text{ind}(i)} - \hat{\theta}_c^{\text{ind}}\right\}\right] \\
&\approx \frac{1}{s^2}\left\{\frac{1}{k}\left(2\hat{\theta}_c^{\text{dir}} + \sum_{i,i\neq d}\hat{\theta}_c^{\text{ind}(i)}\right) - \hat{\theta}_c^{\text{ind}(d)}\right\} \\
&\quad \times \left[\frac{1}{k}\left\{\left(\hat{\theta}_c^{\text{dir}} - \hat{\theta}_c^{\text{ind}}\right) + \left(\hat{\theta}_c^{\text{dir}} - \frac{1}{k}\sum_{i,i\neq d}\hat{\theta}_c^{\text{ind}(i)}\right)\right\}\right]
\end{aligned}$$

Let

$$P_1 \approx \frac{1}{s^2}\left\{\frac{1}{k}\left(2\hat{\theta}_c^{\text{dir}} + \sum_{i,i\neq d}\hat{\theta}_c^{\text{ind}(i)}\right) - \hat{\theta}_c^{\text{ind}(d)}\right\}$$

$$P_2 \approx \frac{1}{k}\left\{\left(\hat{\theta}_c^{\text{dir}} - \hat{\theta}_c^{\text{ind}}\right) + \left(\hat{\theta}_c^{\text{dir}} - \frac{1}{k}\sum_{i,i\neq d}\hat{\theta}_c^{\text{ind}(i)}\right)\right\}.$$

We can simplify P_1 & P_2 further.

$$P_1 \approx \frac{1}{s^2}\left\{\frac{2}{k}\hat{\theta}_c^{\text{dir}} + \frac{1}{k}\sum_{i,i\neq d}\hat{\theta}_c^{\text{ind}(i)} - \hat{\theta}_c^{\text{ind}(d)}\right\}$$

$$P_2 \approx \frac{1}{k}\left\{2\hat{\theta}_c^{\text{dir}} - \hat{\theta}_c^{\text{ind}} - \frac{1}{k}\sum_{i,i\neq d}\hat{\theta}_c^{\text{ind}(i)}\right\}$$

Let $\hat{\theta}_c^{\text{ind}(-d)}$ = average $\left(\sum_{i,i\neq d}\hat{\theta}_c^{\text{ind}(i)}\right)$ then if k is large, $\frac{1}{k}$ is small and

$$P_1 \approx \frac{1}{s^2}\left\{\hat{\theta}_c^{\text{ind}(-d)} - \hat{\theta}_c^{\text{ind}(d)}\right\}$$

$$P_2 \approx \frac{2}{k}\left(\hat{\theta}_c^{\text{dir}} - \hat{\theta}_c^{\text{ind}}\right)$$

C Additional Tables & Figures

Table S1

Results for $Q^{\text{diff}}_{c,d}$, Q^{inc}_{c} and $Q^{\text{inc}}_{c(d)}$ as the number of treatment loops in a network increases (Section 5).

Loops	$Q^{\text{diff}}_{c,d}$	Q^{inc}_{c}	$Q^{\text{inc}}_{c(d)}$
1	42.325	42.325	0.000
2	26.868	26.869	0.001
3	18.430	18.431	0.0004
4	12.910	12.919	0.009
5	10.210	10.210	0.0001
6	8.498	8.507	0.009
7	7.004	7.018	0.014
8	5.874	5.891	0.017
9	4.838	4.846	0.008
10	4.634	4.691	0.057

Table S2

Node splitting results for the diabetes network. Coef = coefficient, Std.Err. = standard error.

	Direct		Indirect		Difference		
Comparison	Coef.	Std. Err.	Coef.	Std. Err.	Coef.	Std. Err.	P-value
acar metf	0.23	0.34	0.29	0.12	-0.06	0.36	0.87
acar plac	-0.82	0.14	-0.84	0.18	0.03	0.23	0.90
acar sulf	-0.40	0.15	-0.37	0.17	-0.03	0.23	0.91
metf piog	-0.16	0.08	0.19	0.12	-0.35	0.15	0.02
metf plac	-1.15	0.08	-1.06	0.09	-0.09	0.12	0.47
metf rosi	0.07	0.16	0.09	0.07	-0.02	0.18	0.92
metf sulf	-0.37	0.12	-1.06	0.13	0.69	0.18	<0.001
piog plac	-1.30	0.13	-0.94	0.09	-0.36	0.16	0.02
piog rosi	0.10	0.18	0.14	0.09	-0.04	0.20	0.83
plac rosi	1.15	0.05	1.47	0.12	-0.32	0.13	0.01
rosi sulf	-1.20	0.14	-0.46	0.12	-0.74	0.19	<0.001

Figure S1

Forest plot of various analyses of the diabetes network. All models were fitted with fixed effects. Key to treatments: acar = acarbose, benf = benfluorex, metf = metformin, migl = miglitol, piog = pioglitazone, plac = placebo, rosi = rosiglitazone, sita = sitagliptin, sulf = sulfonylurea, vild = vildagliptin. CI = confidence interval.

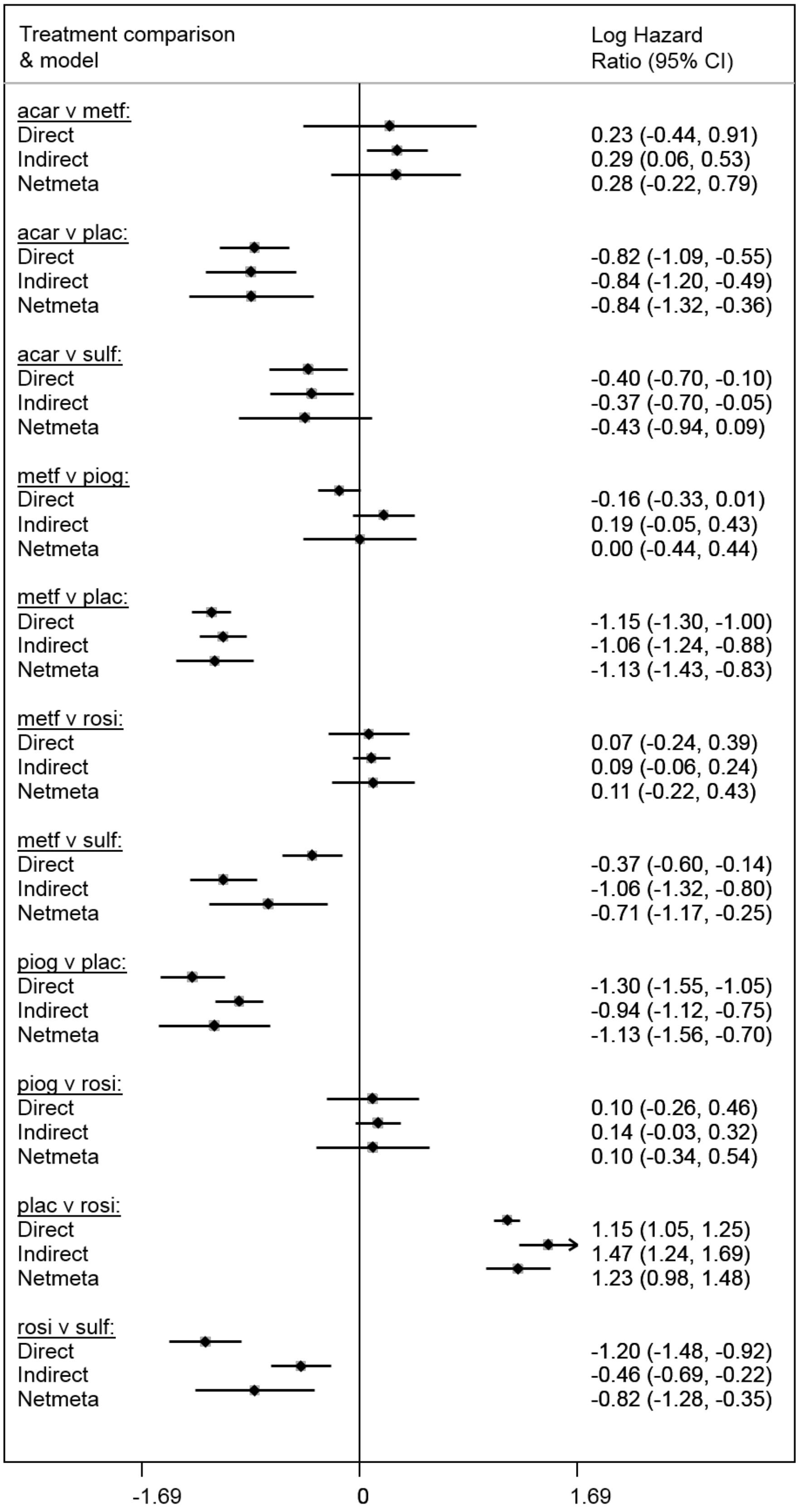

Treatment comparison
& model
Log Hazard
Ratio (95% CI)
acar v metf:
Direct 0.23 (-0.44, 0.91)
Indirect 0.29 (0.06, 0.53)
Netmeta 0.28 (-0.22, 0.79)
acar v plac:
Direct -0.82 (-1.09, -0.55)
Indirect -0.84 (-1.20, -0.49)
Netmeta -0.84 (-1.32, -0.36)
acar v sulf:
Direct -0.40 (-0.70, -0.10)
Indirect -0.37 (-0.70, -0.05)
Netmeta -0.43 (-0.94, 0.09)
metf v piog:
Direct -0.16 (-0.33, 0.01)
Indirect 0.19 (-0.05, 0.43)
Netmeta 0.00 (-0.44, 0.44)
metf v plac:
Direct -1.15 (-1.30, -1.00)
Indirect -1.06 (-1.24, -0.88)
Netmeta -1.13 (-1.43, -0.83)
metf v rosi:
Direct 0.07 (-0.24, 0.39)
Indirect 0.09 (-0.06, 0.24)
Netmeta 0.11 (-0.22, 0.43)
metf v sulf:
Direct -0.37 (-0.60, -0.14)
Indirect -1.06 (-1.32, -0.80)
Netmeta -0.71 (-1.17, -0.25)
piog v plac:
Direct -1.30 (-1.55, -1.05)
Indirect -0.94 (-1.12, -0.75)
Netmeta -1.13 (-1.56, -0.70)
piog v rosi:
Direct 0.10 (-0.26, 0.46)
Indirect 0.14 (-0.03, 0.32)
Netmeta 0.10 (-0.34, 0.54)
plac v rosi:
Direct 1.15 (1.05, 1.25)
Indirect 1.47 (1.24, 1.69)
Netmeta 1.23 (0.98, 1.48)
rosi v sulf:
Direct -1.20 (-1.48, -0.92)
Indirect -0.46 (-0.69, -0.22)
Netmeta -0.82 (-1.28, -0.35)
-1.69
0
1.69

D R code

R code for Section 5

```
# Load package
library(netmeta)

# 10 TREATMENT LOOPS

n.in.design<-6 # number of trials per design
n.design<-21 # number of designs

trialid <- seq(1, n.in.design*n.design, 1)

trt1 <- c(rep("A", n.in.design), rep("A", n.in.design), rep("B", n.in.design),
          rep("A",n.in.design),rep("B",n.in.design),
          rep("A",n.in.design),rep("B",n.in.design),
          rep("A",n.in.design),rep("B",n.in.design),
          rep("A",n.in.design),rep("B",n.in.design),
          rep("A",n.in.design),rep("B",n.in.design),
          rep("A",n.in.design),rep("B",n.in.design),
          rep("A",n.in.design),rep("B",n.in.design),
          rep("A",n.in.design),rep("B",n.in.design),
          rep("A",n.in.design),rep("B",n.in.design)
)



trt2 <- c(rep("B", n.in.design), rep("C", n.in.design), rep("C", n.in.design),
          rep("D",n.in.design), rep("D",n.in.design),
          rep("E",n.in.design), rep("E",n.in.design),
          rep("F",n.in.design), rep("F",n.in.design),
          rep("G",n.in.design), rep("G",n.in.design),
```

```
          rep("H",n.in.design), rep("H",n.in.design),
          rep("I",n.in.design), rep("I",n.in.design),
          rep("J",n.in.design), rep("J",n.in.design),
          rep("K",n.in.design), rep("K",n.in.design),
          rep("L",n.in.design), rep("L",n.in.design)
)

set.seed(10)
within.design.sd=0.2
te <- c(rnorm(n.in.design,mean=0,sd=within.design.sd),
        rnorm(n.in.design,mean=2,sd=within.design.sd),
        rnorm(n.in.design,mean=0,sd=within.design.sd),
        rnorm(n.in.design,mean=0,sd=within.design.sd),
        rnorm(n.in.design,mean=0,sd=within.design.sd),
        rnorm(n.in.design,mean=0,sd=within.design.sd),
        rnorm(n.in.design,mean=0,sd=within.design.sd),
        rnorm(n.in.design,mean=0,sd=within.design.sd),
        rnorm(n.in.design,mean=0,sd=within.design.sd),
        rnorm(n.in.design,mean=0,sd=within.design.sd),
        rnorm(n.in.design,mean=0,sd=within.design.sd),
        rnorm(n.in.design,mean=0,sd=within.design.sd),
        rnorm(n.in.design,mean=0,sd=within.design.sd),
        rnorm(n.in.design,mean=0,sd=within.design.sd),
        rnorm(n.in.design,mean=0,sd=within.design.sd),
        rnorm(n.in.design,mean=0,sd=within.design.sd),
        rnorm(n.in.design,mean=0,sd=within.design.sd),
        rnorm(n.in.design,mean=0,sd=within.design.sd),
        rnorm(n.in.design,mean=0,sd=within.design.sd),
        rnorm(n.in.design,mean=0,sd=within.design.sd),
        rnorm(n.in.design,mean=0,sd=within.design.sd),
        rnorm(n.in.design,mean=0,sd=within.design.sd)

)
```

```
set.seed(123)
sete <- dnorm(te)

data.nh <- data.frame(studlab=trialid, treat1=trt1, treat2=trt2, TE=te, seTE=sete)

# Conduct fixed and random effects NMA using A as reference group
nm <- netmeta(TE=TE, seTE=seTE, treat1=treat1, treat2=treat2, studlab=trialid,
data=data.nh, comb.fixed=TRUE,  reference.group="A", sm="MD")

# Check network diagram
netgraph(nm)

# Check results
summary(nm)

# net heat
netheat(nm)

# EXTRACT Qdiff

nmak <- netmeta:::nma.krahn(nm)
V <- nmak$V

# Decompose the nm model
# This gives us Qnet, Qhet and Qinc statistics
decomp <- decomp.design(nm)

# Next we create a matrix containing the residuals
residuals <- decomp$residuals.inc.detach

# Qinc statistics by design
```

```
Q.inc.design <- decomp$Q.inc.design

# Calculate Qinc(d'(d))
Q.inc.design.typ <- apply(residuals, 2, function(x) t(x) %*%
                            solve(V) * x)

# Matrix of Qinc(d') statistics
inc <- matrix(Q.inc.design, nrow = nrow(Q.inc.design.typ),
              ncol = ncol(Q.inc.design.typ))

#Calculate Qdiff(d',d)
diff <- inc - Q.inc.design.typ

# Vector of column names (i.e. the comparisons)
colnames(diff) <- colnames(Q.inc.design.typ)

# Vector of row names (i.e. the comparisons)
rownames(diff) <- rownames(residuals)

# Matrix of Qdiff(d',d)
diff

# Save Qdiff value for row=AB and column=AC
qdiff[10] <- diff[1,2]
qinc[10] <- inc[1,2]
qincdesign[10] <- Q.inc.design.typ[1, 2]
```
